# Reliability of pair distribution function analysis in *in situ* experiments

**DOI:** 10.1107/S1600576725001694

**Published:** 2025-03-19

**Authors:** Rasmus Baden Stubkjær, Magnus Kløve, Andreas Bertelsen, Anders Bæk Borup, Martin Roelsgaard, Bo Brummerstedt Iversen

**Affiliations:** a Department of Chemistry and Interdisciplinary Nanoscience Center (iNANO) Aarhus University Langelandsgade 140 Aarhus8000-DK Denmark; The University of Western Australia, Australia

**Keywords:** pair distribution functions, *in situ* studies, nanocrystal nucleation, nanocrystal growth, time-resolved experiments

## Abstract

*In situ* and *operando* pair distribution function (PDF) studies are becoming commonly used to study chemical reactions, nucleation and growth of nanoparticles, or structural changes during the operation of batteries, catalysts, thermoelectric devices *etc.* However, repeated time-resolved total scattering experiments and subsequent PDF analysis are often not prioritized due to the scarce synchrotron beam time available. This means that the experimental uncertainty and reproducibility of the experimental methods are unknown, and the full potential of *in situ* PDF experiments may not be exploited. Here, we quantify the experimental uncertainty of the PDF technique in an *in situ* study of the hydro­thermal synthesis of ZrO_2_ nanoparticles. Systematic variation of the parameters used to obtain the PDF shows that the user-defined parameters can potentially affect the chemical conclusions obtained from the time-resolved experiment. We found that comparable results are best obtained using the same input parameters across different experiments. We also compare different PDF algorithms to examine whether the processing algorithm influences the chemical analysis.

## Abstract 2

The reproducibility of *in situ* pair distribution function studies of solvothermal reactions is investigated through detailed analysis of repeated measurements.

## 1. Introduction

Designing new functional materials with optimized properties relies on the ability to tailor their structural characteristics. To successfully design such specific characteristics, for example the size, shape, morphology and phase of a nanosized catalyst, control of the synthesis route is necessary (Saha *et al.*, 2014 ▸; Birgisson *et al.*, 2018 ▸; Cuenya, 2010 ▸; Joo *et al.*, 2019 ▸; Quinson *et al.*, 2018 ▸). The use of *in situ*X-ray scattering experiments is a time-efficient and valuable method for obtaining structural insight during the synthesis of different materials (Hatchard & Dahn, 2004 ▸; Jensen *et al.*, 2007 ▸; Baylet *et al.*, 2011 ▸; Hesse *et al.*, 2011 ▸; Jensen *et al.*, 2014 ▸). The ability to follow structural changes during synthesis allows for the observation of metastable intermediates as well as tracking characteristics such as particle size, strain, crystallinity, defects *etc.* (Norby, 2006 ▸; Tyrsted *et al.*, 2014 ▸; Mi *et al.*, 2015 ▸; Zobel *et al.*, 2016 ▸; Prinz *et al.*, 2023 ▸).

Analysis of time-resolved X-ray scattering experiments is often performed in reciprocal space using sequential Rietveld refinements. Such analysis allows for an atomistic description of the observed reaction mechanism (Bremholm *et al.*, 2009 ▸; Oezaslan *et al.*, 2011 ▸; Zheng *et al.*, 2023 ▸; Quinson & Jensen, 2020 ▸). The analysis relies on distinct Bragg reflections, which are a result of the formation of crystalline domains. However, in some cases non-crystalline intermediates or products are formed which complicate the use of Rietveld refinements. An example is found in the case of Ir (Mathiesen *et al.*, 2023 ▸) or Ir_1−*x*_Ru_*x*_O_2_ nanoparticle synthesis (Bertelsen *et al.*, 2024 ▸), where <2 nm particles are formed yielding severe broadening in reciprocal space. Another case is the sodium discharge from iron(III) hydroxide phosphate hydrate, where an amorphous intermediate forms (Henriksen *et al.*, 2020 ▸). Similarly, an amorphous intermediate forms prior to crystallization in solvothermal synthesis of ZrO_2_ (Tyrsted *et al.*, 2014 ▸). To obtain atomistic insight into non-crystalline or borderline-crystalline phases (*e.g.* nanoparticles), the total scattering (TS) pattern can be analyzed in direct space by calculating the pair distribution function (PDF) (Egami & Billinge, 2012 ▸).

The combination of time-resolved X-ray TS experiments and PDF analysis of nanoparticle formation was introduced in 2012 in studies of SnO_2_ (Jensen *et al.*, 2012 ▸) and CeO_2_ (Tyrsted *et al.*, 2012 ▸) and has since been used for a variety of different synthesis methods including solvothermal (Greenberg *et al.*, 2023 ▸; Juelsholt *et al.*, 2023 ▸), sol–gel (Chambers *et al.*, 2021 ▸; Morandeau & White, 2015 ▸), microwave radiation assisted (Nakamura *et al.*, 2020 ▸) and pyrolysis (Frank *et al.*, 2024 ▸). When attempting to synthesize nanoparticles, solvothermal conditions are often used, since this enables fast, cost-effective, green and easily scalable synthesis (Aymonier *et al.*, 2018 ▸). A reactor setup has been under continuous development over the past 20 years in our group to study these solvothermal reactions *in situ* using X-ray TS techniques (Becker *et al.*, 2010 ▸). This setup is now a part of the instrument pool at selected beamlines at the PETRA III and MAX IV synchrotrons (Roelsgaard *et al.*, 2023 ▸).

Accounting for the reproducibility of observed phenomena is a foundation of the natural sciences. Standardized experimental procedures for obtaining reproducible results have been developed within different scientific fields such as the preparation of battery cells (Dai & Cai, 2022 ▸), reporting electrocatalytic performance (Voiry *et al.*, 2018 ▸), investigating the stability of thermoelectric materials (Jørgensen & Iversen, 2022 ▸) or collecting quantitative scanning electron microscopy–energy dispersive X-ray spectroscopy data (Newbury & Ritchie, 2013 ▸). In the case of *in situ* TS studies, multiple repetitions using similar experimental conditions are often not performed due to the scarcity of the available synchrotron beam time. Although each acquired scattering pattern can be regarded as an independent measurement, the chemical mechanisms derived from the entirety of the experiment should only be considered as a single observation. Benchmarking of the experimental setup and mapping out of pitfalls in the data analysis procedure are thus necessary to validate observations.

The pitfalls and reproducibility of powder X-ray diffraction (PXRD) studies of *in situ* solvothermal reactions were studied in detail by Andersen *et al.* (2018 ▸), who concluded that the focus in these types of experiments should be on relative trends rather than absolute values. Here, we report on the reproducibility of *in situ* solvothermal synthesis studied using X-ray TS and PDF analysis. In addition, we map out the influence of user input during the data reduction and develop a ‘best practice’ for comparing time-resolved PDF studies.

## 2. Methods

The hydro­thermal syntheses were performed using a 1 *M* ZrCl_4_ (99.5% Alfa Aesar) solution in MilliQ H_2_O. The reactions were carried out in a 0.7 mm inner diameter fused silica capillary pressurized to ∼250 bar and heated directly to 250 °C. The experimental setup is described in detail by Roelsgaard *et al.* (2023 ▸). The temperature calibration is further described in Figs. S1 and S2 of the supporting information.

Ten experiments were performed at beamline P21.1, DESY, Hamburg, using the same stock solution as the precursor. The beamline was operated using a beam energy of 101.46 keV (0.1222 Å). TS data were collected using a Pilatus3 X CdTe 2M detector with an exposure time of 1 s. TS data were also collected for the standard reference material (SRM) LaB_6_ NIST 660b for calibration. To assess the quality of the calibration, ten measurements were performed in the beam time. In addition, one experiment was performed using similar conditions at the DanMAX beamline, MAX IV, Sweden, with a beam energy of 35.00 keV (0.35424 Å). The additional experiment conducted at DanMAX was necessary for processing the data in *GudrunX*, as no scattering pattern from the empty instrument was acquired at P21.1 but one is required for the *GudrunX* algorithm.

The TS data were azimuthally averaged using *pyFAI*, with 3000 azimuthal bins. Calibration and masking were performed using *pyFAI-calib2* (Kieffer *et al.*, 2020 ▸). Unless explicitly specified, the PDFs were calculated using the *PDFgetX3* algorithm (Juhás *et al.*, 2013 ▸) with a composition of ZrO_2_. The background correction was done using TS data from a capillary filled with H_2_O pressurized and heated to ∼250 bar and 250 °C. The PDFs were calculated using varying parameters; however, only one parameter was varied at a time. The baseline parameters for the other PDFs to be compared against were *Q*_max_ = 18 Å^−1^, *R*_poly_ = 0.9 Å, *Q*_min_ = 1 Å^−1^ and *Q*_max,inst_ = 22 Å^−1^.

The PDFs obtained from the TS dataset collected at DanMAX were processed differently to compare *GudrunX* (Soper & Barney, 2011 ▸), *PDFgetX3* (Juhás *et al.*, 2013 ▸) and *TOPAS* (version 7; Coelho, 2018 ▸). Examples of the input files are provided in Sections S14–16 of the supporting information. A composition of (H_2_O)_55.6_ZrCl_4_ was assumed to describe the 1 *M* ZrCl_4_ aqueous solution. The background correction was done using TS data of an empty capillary heated to 250 °C. The PDFs were calculated using *Q*_max_ = 16.5 Å^−1^ and *Q*_min_ = 1 Å^−1^. The change in composition and background subtraction was necessary to calculate the PDFs using *GudrunX*.

The main difference between the three algorithms is the correction of incoherent scattering. In *GudrunX*, the incoherent scattering correction is based on scaled table values using the method developed by Krogh-Moe (1956 ▸) and Norman (1957 ▸), whereas an *ad hocR*_poly_ correction is used in *PDFgetX3* (Billinge & Farrow, 2013 ▸). *TOPAS* allows for user-defined macros, and in the current algorithm a sixth-degree Chebyshev polynomial weighted by *Q*/*Q*_max_ is used resembling the *ad hocR*_poly_ correction of *PDFgetX3*.

All sequential refinements of the PDFs were performed in *TOPAS* (version 7). The refined model was based on the crystal structure of ZrO_2_ reported by Gualtieri *et al.* (1996 ▸) (ICSD No. 82544). The scale factor, lattice parameters, isotropic atomic displacement parameters (ADPs) and spherical radius were refined.

## 3. Results

### 3.1. Experimental uncertainty

**Figure 1:**
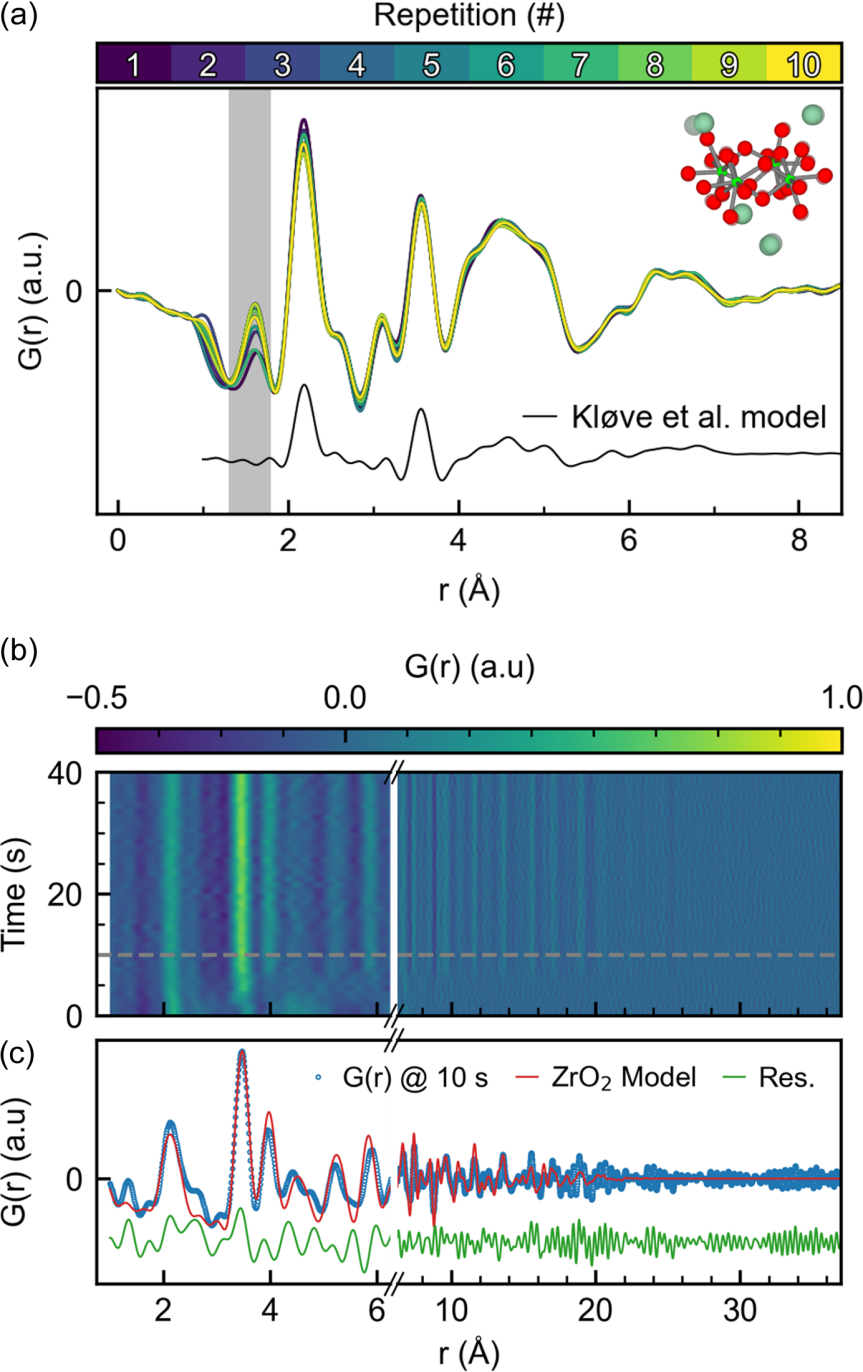
(*a*) Variation in the observed precursor PDF across all ten repetitions. The black line is a simulation of the model proposed by Kløve *et al.* (2022 ▸) and the structure inset is a visual representation of the precursor solution structure (O: red; Zr: light green; Cl: dark green). (*b*) 2D contour plot of the PDFs obtained for the first 40 s after heat is applied. The gray dashed line marks 10 s. (*c*) Fit of the PDF after 10 s. Note that (*b*) and (*c*) are constructed with a broken axis to highlight the low-*r* region.

To evaluate the experimental uncertainty of the hydro­thermal synthesis of ZrO_2_ from ZrCl_4_ in H_2_O, a total of ten experiments were performed, referred to as rep. 1–10. Fig. 1 ▸(*a*) shows the variation in the PDF of the precursor for rep. 1–10, and the corresponding reduced structure function, *F*(*Q*), is shown in Fig. S3. A Pearson correlation analysis (Fig. S4) shows minimal variation between the room-temperature PDFs, indicating that the run-to-run variation is low for these types of experiments. Furthermore, the obtained PDFs agree well with the model proposed by Kløve *et al.* (2022 ▸). The additional peak observed at ∼1.6 Å (shaded area) is a result of insufficient background subtraction. The fact that very similar structural motifs are observed for the ten precursor solutions indicates that the initial starting point of each reaction is comparable.

A 2D contour plot of the reaction observed in rep. 1 is shown in Fig. S5, and Fig. 1 ▸(*b*) shows a contour plot of the corresponding PDFs for the first 40 s. ZrO_2_ forms within ∼10 s after heating is initiated in all ten repetitions, apparent from the *F*(*Q*) at ∼10 s shown in Fig. S6. Fig. 1 ▸(*c*) shows the PDF of rep. 1 obtained after 10 s and the subsequent refined monoclinic model. The monoclinic ZrO_2_ phase forms directly from the ZrCl_4_ solution, which is different from the hydro­thermal reaction of other zirconium precursors (Dippel *et al.*, 2016 ▸).

**Figure 2:**
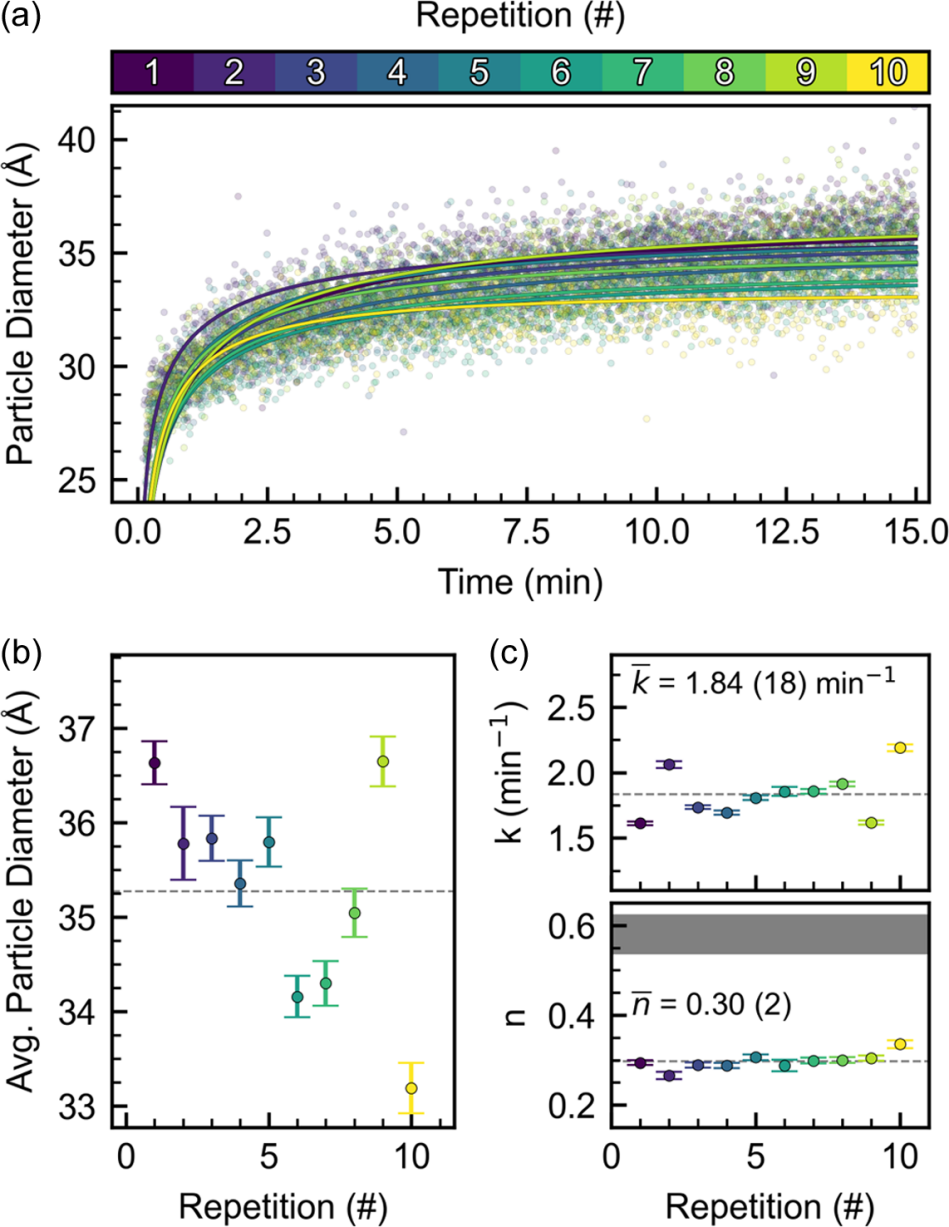
(*a*) Particle diameter obtained from sequential refinements. Fitted growth models [equation (1)] are plotted on top of each dataset. (*b*) Final particle diameter obtained by averaging the final 60 frames of each repetition. The gray dashed line illustrates the average particle diameter (35.3 Å) across all ten repetitions. The error bars correspond to the mathematical standard uncertainty obtained from the least-squares minimization. (*c*) Resulting growth model parameters *k* and *n* obtained for each repetition.

Figs. 2 ▸(*a*) and S7 show the particle diameter with time for all ten repetitions. The particle diameter reaches 30 Å within the first minute with a small variation among the repetitions. Further growth leads to a final average diameter of 35.3 (11) Å across the ten experiments shown in Fig. 2 ▸(*b*). The observed standard deviation of the particle diameter corresponds to a variation of 3%, which is roughly half the uncertainty of 1 nm for the 15 nm Fe_2_O_3_ particles (∼6.7%) found by Andersen *et al.* (2018 ▸). Their study used an earlier version of the experimental setup employed here for a similar analysis of *in situ* PXRD patterns. The observed decrease in experimental uncertainty is assumed to be a result of an improved mounting procedure and the implementation of a new heating source.

By visual inspection of Fig. 2 ▸(*a*), the refined particle diameter follows the same trend across the ten repetitions. This is a consequence of a robust experimental setup with precise sample positioning and control over experimental parameters across multiple days of beam time. To obtain a qualitative measure of whether the chemical reaction observed is comparable across the ten repetitions, a growth curve is fitted for each experiment in Fig. 2 ▸(*a*). The applied kinetic model is described in equation (1) and is based on classical nucleation and growth theory following Johnson & Mehl (1939 ▸), Avrami (1939 ▸) and Kolmogorov (1937 ▸).
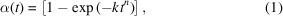
where α is the extent of reaction, *k* is the rate constant related to crystal nucleation and growth, and *n* is related to the reaction mechanism. As an example, *n* values of ∼0.6 have been linked to a diffusion-controlled mechanism (Hancock & Sharp, 1972 ▸). The rate constant (*k*) is plotted in Fig. 2 ▸(*c*) together with the *n* parameter. Fig. 2 ▸(*c*) shows that neither *k* nor *n* changes significantly across the ten repetitions. An average rate constant of 1.84 (18) min^−1^ is determined across all ten repetitions, along with an average *n* value of 0.30 (2). The analysis shows that reproducible reaction kinetics are obtained across the ten experiments; however, further analysis is needed to draw definitive conclusions regarding the nucleation and growth processes, which is beyond the scope of this study.

**Figure 3:**
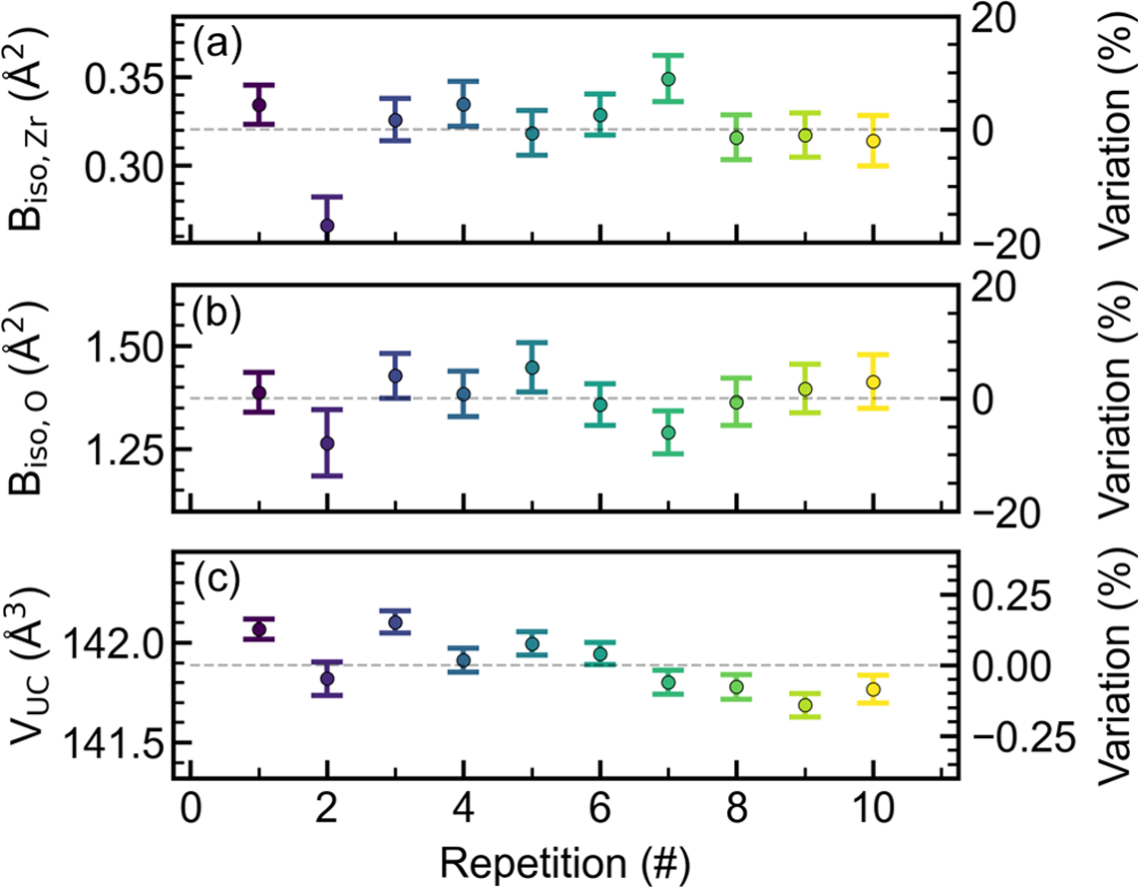
Variation of refined parameters during the final minute of the experiment [(*a*) B_iso,Zr_, (*b*) B_iso,O_, (*c*) unit-cell volume] for each repetition compared with the average value across all ten repetitions. The error bars correspond to the mathematical standard uncertainty obtained from the least-squares minimization.

In addition to the particle diameter used for kinetic analysis, the isotropic ADPs and the unit-cell parameters are also obtained in the sequential refinement of the PDFs (Fig. S8). After nucleation the unit-cell parameters and the ADPs do not change significantly (Fig. S8). The final values obtained for the ADPs vary on the order of ∼20% across the ten repetitions, whereas the variation observed for the unit cell is on the order of 0.2% (Fig. 3 ▸). Thus, the unit-cell volume is well determined with a refined volume of 141.9 ± 0.3 Å^3^, similar to previously reported values (Gualtieri *et al.*, 1996 ▸; Dippel *et al.*, 2016 ▸). It is known that the absolute ADPs in the PDF are handled incorrectly for materials consisting of more than one atomic species in most small-box modeling software, as the *Q* dependence of the atomic scattering factors is neglected to simplify the calculation (Neder & Proffen, 2020 ▸). This could influence the large variations observed for the ADPs for ZrO_2_. Hence care must be taken when interpreting the ADPs during an *in situ* experiment of this type. The description of the ADPs could potentially be improved by introducing methods similar to the one suggested by Neder & Proffen (2020 ▸) into available small-box modeling software.

**Figure 4:**
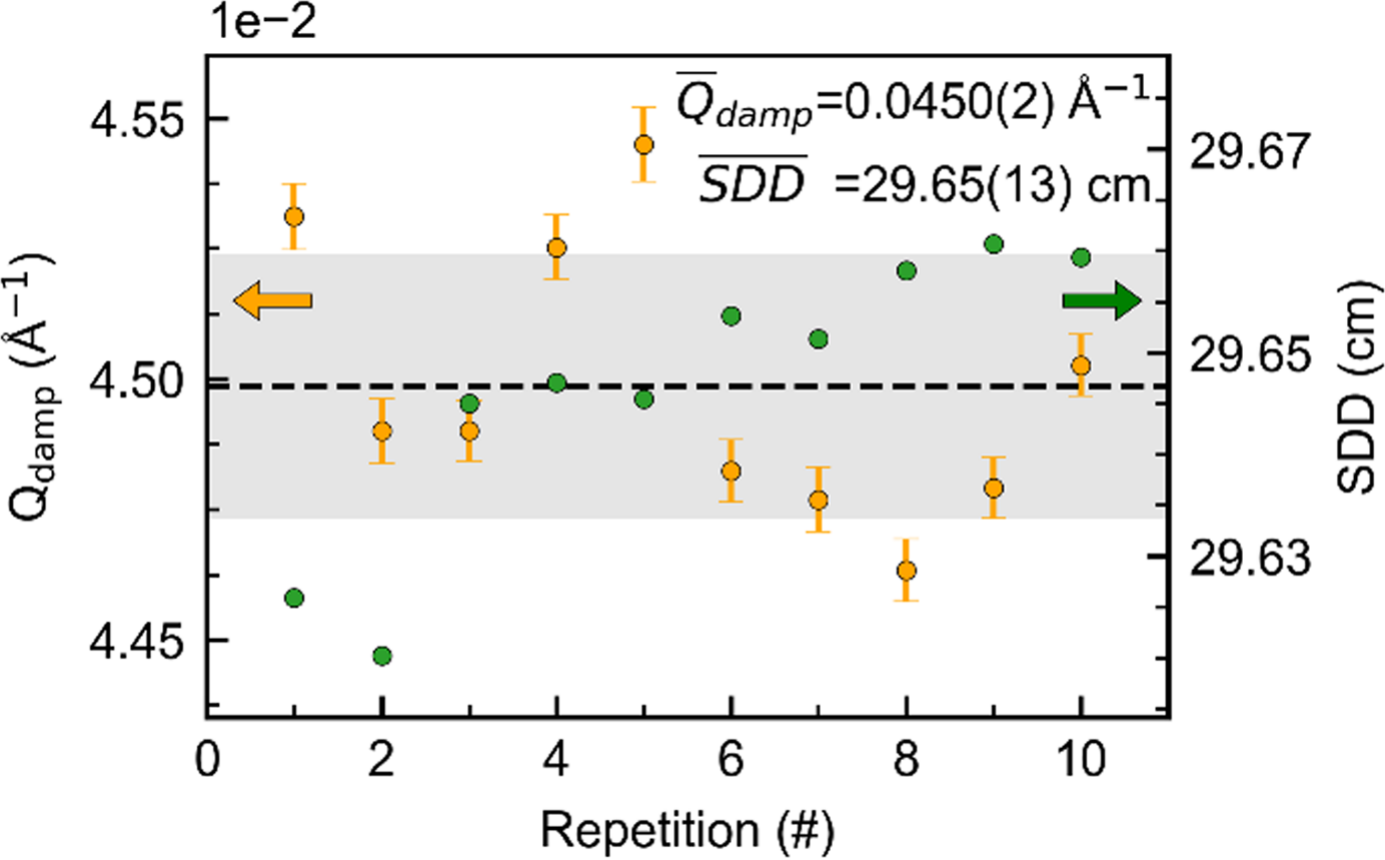
Instrumental *Q*_damp_ and SDD obtained from ten repetitions on LaB_6_ NIST 660b. The gray shaded area highlights the standard deviation for both *Q*_damp_ and SDD, and the dashed line corresponds to the average value. The least-squares error of the SDD is not provided by the *pyFAI* algorithm and therefore cannot be reported.

The total variation observed across the ten repetitions is a result of different contributions, which can be divided into two categories: those originating from the experiment (*e.g.* sample mounting, integration, post-processing *etc.*) and those originating from the differences in observed phenomena. To assess the errors introduced by the experimental setup, the TS from an LaB_6_ NIST 660b sample was collected ten times across the beam time. The Gaussian experimental dampening, *Q*_damp_, and the sample-to-detector distance (SDD) obtained from the ten repetitions are shown in Fig. 4 ▸.

*Q*_damp_ is directly related to the refined particle diameter. Thus, a stable *Q*_damp_ across the beam time is paramount when comparing particle sizes. The average 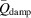 is determined to be 0.0450 (2) Å^−1^ across the ten LaB_6_ measurements. The SDD (Fig. 4 ▸) will directly affect the unit-cell dimensions. The deviations observed are a measure of the sample-mounting accuracy. The 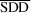
 = 29.65 (1) cm obtained from the ten experiments can be directly compared with the analysis done by Andersen *et al.* (2018 ▸), who obtained 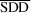
 = 8.99 (7) cm. The sevenfold improved accuracy in the SDD is mainly due to further development of the experimental setup and beamline upgrades (Roelsgaard *et al.*, 2023 ▸).

**Figure 5:**
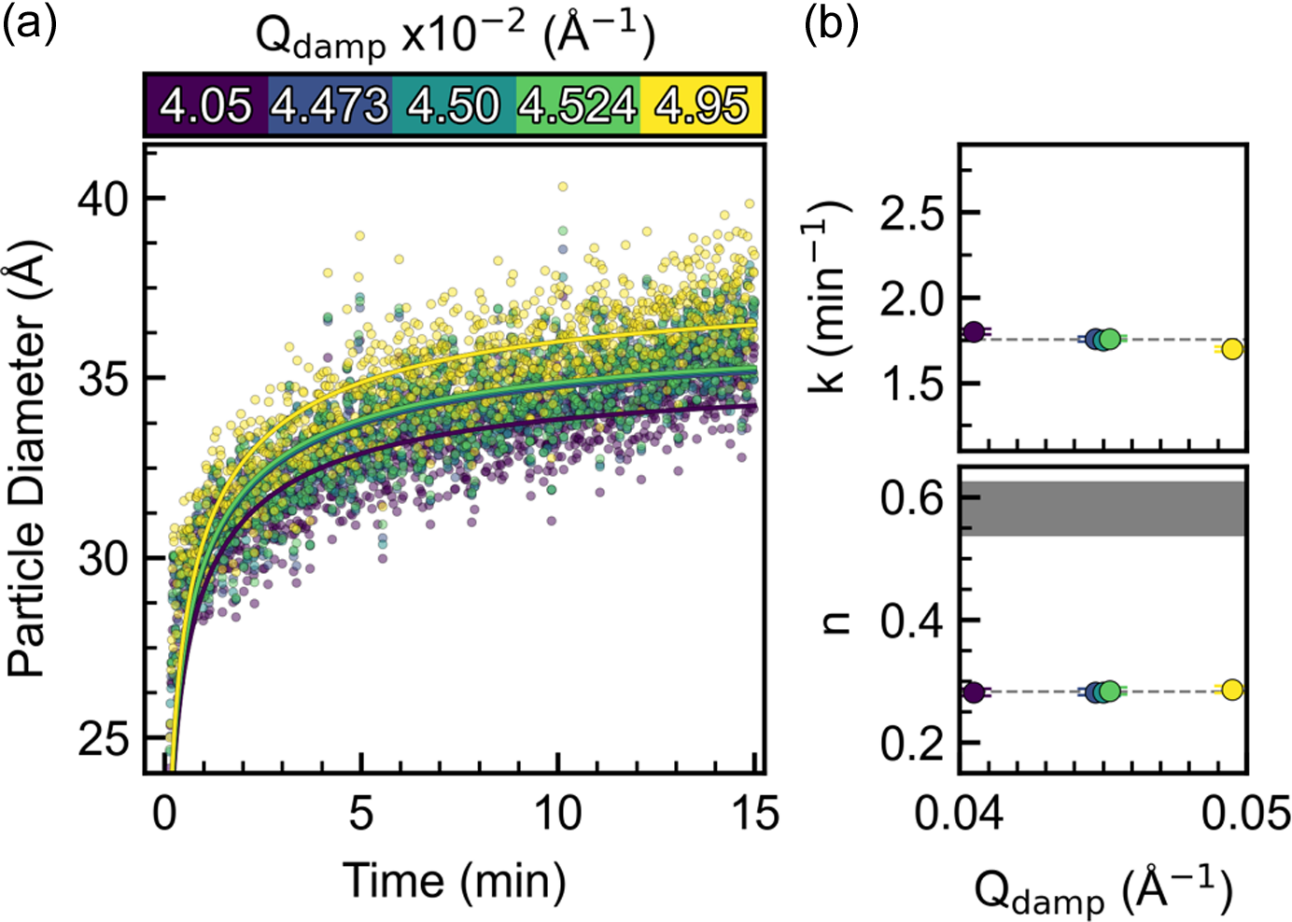
(*a*) Particle diameter obtained from systematically varying the *Q*_damp_ value between 0.0405 and 0.0495 Å^−1^ with (*b*) *k* and *n* from the corresponding growth model fits.

Sequential refinements using varying *Q*_damp_ values were performed, and the particle diameters obtained are plotted in Fig. 5 ▸(*a*). The value of 0.0450 Å^−1^ corresponds to the average found in Fig. 4 ▸ and the other four values are chosen to correspond to introducing an error of one standard deviation (0.04473 and 0.04524 Å^−1^) or introducing an error of 10% (0.0405 and 0.0495 Å^−1^). The variation in *Q*_damp_ can be directly related to a variation in the final particle diameter. Introducing an error of the *Q*_damp_ value corresponding to one standard deviation (0.002 Å^−1^) leads to an error in the particle diameter of ∼0.1 Å (Fig. S9). Additional contributions to the experimental error on the particle diameter such as a consistent heating profile are difficult to estimate in absolute terms, but a substantial contribution is expected. Importantly, the conclusion obtainable from the kinetic analysis seems unaltered using *Q*_damp_ values within one standard deviation [Fig. 5 ▸(*b*)]. In fact, the *Q*_damp_ value seems to have no influence on the time evolution of the other refined values, except for the particle diameter (Fig. S10).

### 3.2. Influence of user inputs

Reducing a TS dataset from raw scattering patterns to an interpretable PDF requires careful data treatment, which relies heavily on user input. Inputs such as *Q*_max_, *Q*_min_, *R*_poly_, background scale *etc.* are all individually selected by the user and often evaluated by visual inspection of the PDF. How – or whether – these user inputs influence the resulting chemical conclusion has not been rigorously studied for time-resolved TS experiments such as the experiments presented here.

**Figure 6:**
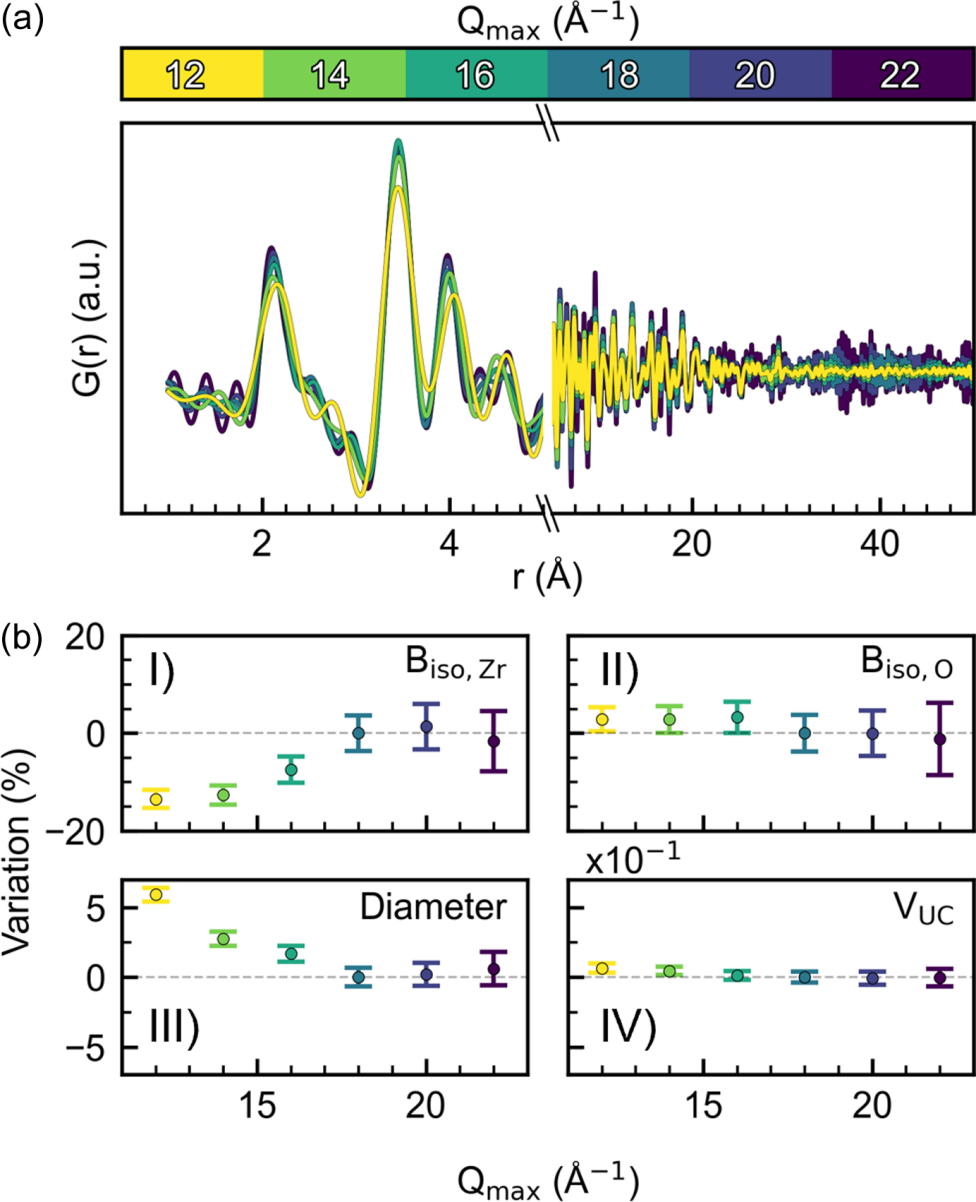
(*a*) Final PDF obtained from the *in situ* experiment after 15 min using varying *Q*_max_ values from 12 to 22 Å^−1^. (*b*) Variation in refined parameters (I: B_iso,Zr_; II: B_iso,O_; III: particle diameter; IV: unit-cell volume) within the last 60 s of the *in situ* experiment using varying *Q*_max_ values. The dashed gray line is the values obtained using *Q*_max_ = 18 Å^−1^. The error bars correspond to the mathematical standard uncertainty obtained from the least-squares minimization.

The user inputs *Q*_max_, *Q*_min_, *R*_poly_, background scale and pixel binning were all systematically investigated, and the results are shown in Figs. S11–S24. Some general highlights are presented here. The *Q*_max_ values were varied between 12 and 22 Å^−1^, and the PDFs obtained are shown in Fig. 6 ▸(*a*). Changing *Q*_max_ influences the real space resolution of the PDF, δ*r*, as δ*r* = π/*Q*_max_, the Nyquist–Shannon sampling theorem (Farrow *et al.*, 2011 ▸). This infers that increasing *Q*_max_ results in a sharpening of the PDF peaks; however, including the high scattering angles in the Fourier transform also introduces additional noise due to the lower signal-to-noise ratio at high *Q*. In contrast, a small *Q*_max_ broadens the PDF peaks while unnecessary noise is minimized. *Q*_max_ should thus be optimized depending on the type of analysis required. This is apparent from the variation of the final average values obtained when using different *Q*_max_ where, for example, the particle size decreases with increasing *Q*_max_ until 18 Å^−1^, whereafter the value converges [Fig. 6 ▸(*b*)]. Thus, a reliable particle size is obtained in these experiments with *Q*_max_ = 18 Å^−1^, whereas higher data resolution does not further contribute to this estimate. The same sort of reasoning applies to all the refined parameters. Note that this *Q*_max_ trend depends on the experimental setup, the temperature and the specific chemical system under study. Interestingly, using a small *Q*_max_ leads to an offset in absolute values, but with decreasing standard deviation. The decrease in standard deviation is a result of limiting the signal-to-noise ratio, as evident from the reduced structure function, *F*(*Q*), in Fig. S15.

**Figure 7:**
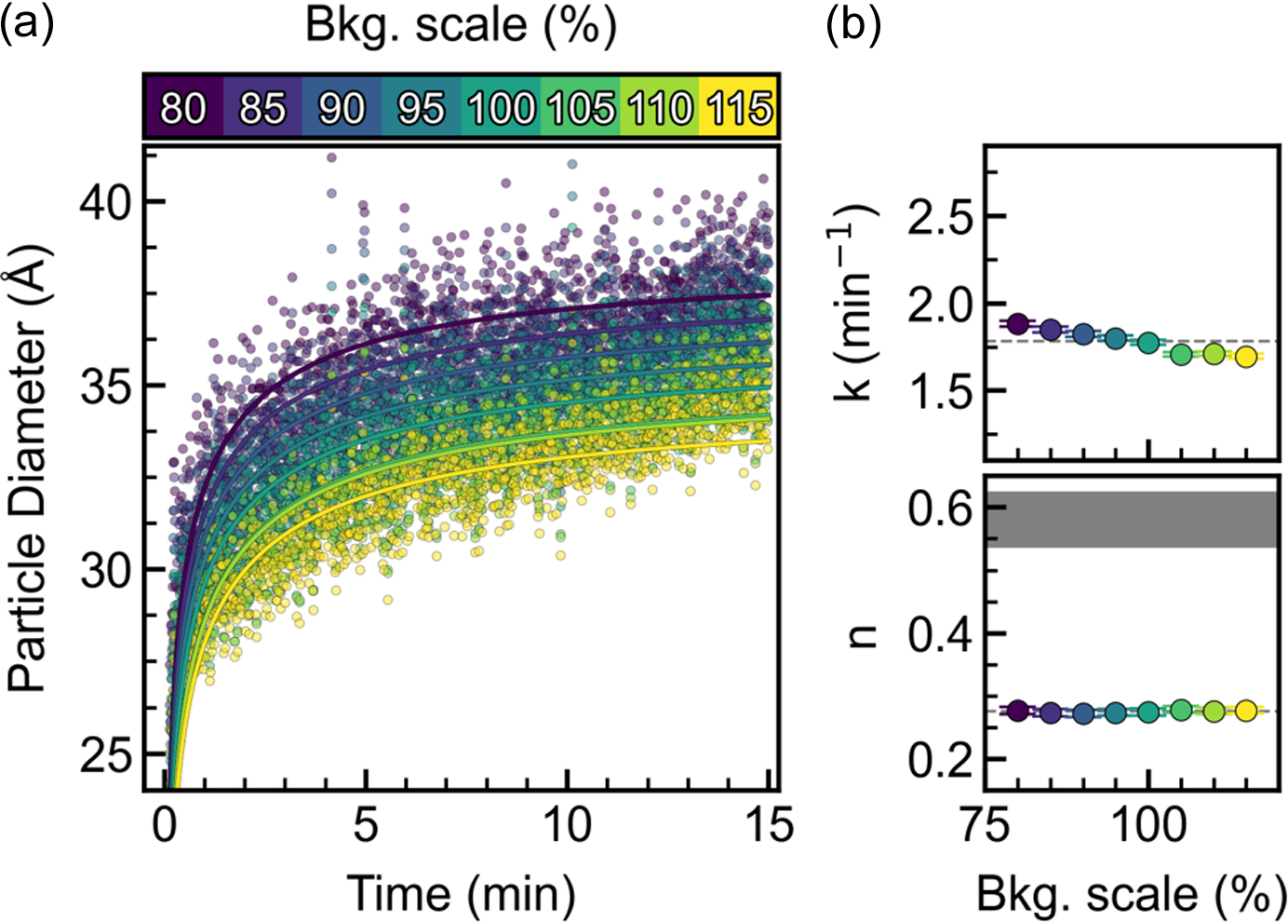
(*a*) Refined particle diameters using varying background subtraction. (*b*) Corresponding growth model results.

Obtaining an interpretable PDF requires correcting the TS pattern for any additional coherent scattering not originating from the sample. This correction is handled differently depending on the algorithm used; however, with the *PDFgetx3* algorithm this is done by scaling an independent scattering pattern from the sample container. Systematically varying the background scale leads to an offset of all absolute values (Figs. S11 and S12), with Fig. 7 ▸(*a*) showing the offset in the particle size. The offset in all refined parameters highlights the fact that the absolute values obtained are difficult to interpret for experiments such as these. Small variations in the background are expected during an experiment, due to solvent changes and temperature fluctuations, especially during heating and cooling. The time dependence was again analyzed using the growth model [equation (1)] and no significant changes are observed for *n*. *k*, on the other hand, decreases linearly with increasing background [Fig. 7 ▸(*b*)] due to the linearly decreasing crystallite size.

Systematic variation of *Q*_min_ was found to significantly influence the refined ADPs when *Q*_min_ values above 1 Å^−1^ were used (Fig. S18). No systematic trends are observed when varying *R*_poly_ (Figs. S19–S21), but importantly some variation is observed, indicating that equal *R*_poly_ values should be used when comparing datasets. Regarding data binning during the azimuthal averaging of the 2D detector image, the refined parameters are not affected significantly until the number of bins approaches a lower limit (between 500–1000 bins in this case, Figs. S22–S24). This is expected as further reducing the number of bins across this point will lead to broadening in reciprocal space. The number of suitable bins is thus also dependent on the nature of the scattering pattern, for example, on the instrumental resolution function or the size of the crystallites.

The composition used during the data reduction process also affects the PDFs obtained (Fig. S25). The composition primarily influences the scale of the PDFs, but changes in the absolute values of the ADPs and the particle sizes are also expected. In the present *in situ* experiments, the composition is expected to change during the experiment, and investigating this effect in detail would require a secondary *in situ* probe such as X-ray florescence spectroscopy.

In general, systematically changing the user input parameters highlights that the applied data processing parameters can affect the chemical conclusions drawn. To compare individual experiments, similar input parameters are required. The analysis also demonstrates that absolute values are prone to error, hence highlighting the fact that systematic trends should be the focus.

### 3.3. Data reduction algorithms

The data reduction shown so far was performed using the *PDFgetX3* algorithm (Juhás *et al.*, 2013 ▸), but other algorithms are available such as *GudrunX* (Soper & Barney, 2011 ▸), *GSAS-II* (Toby & Von Dreele, 2013 ▸), *LiquidDiffract* (Heinen & Drewitt, 2022 ▸) and *TOPAS* (version 7; Coelho, 2018 ▸). The *PDFgetX3* algorithm was chosen in the present study as the Python integration allows for easy batch reduction of thousands of scattering patterns. To review whether the choice of algorithm affects the conclusions of the hydro­thermal reaction, data reduction was performed using *GudrunX*, *PDFgetX3* and *TOPAS* for the experiment conducted at DanMAX. One of the main differences between the three algorithms is the correction for incoherent scattering contributions. In *GudrunX*, this is done using scaled table values dependent on the input composition, whereas the correction performed in *PDFgetX3* is an *ad hoc* correction. The reduction performed in *TOPAS* is similar to the *PDFgetX3* algorithm, but with a slightly modified *ad hoc* correction.

**Figure 8:**
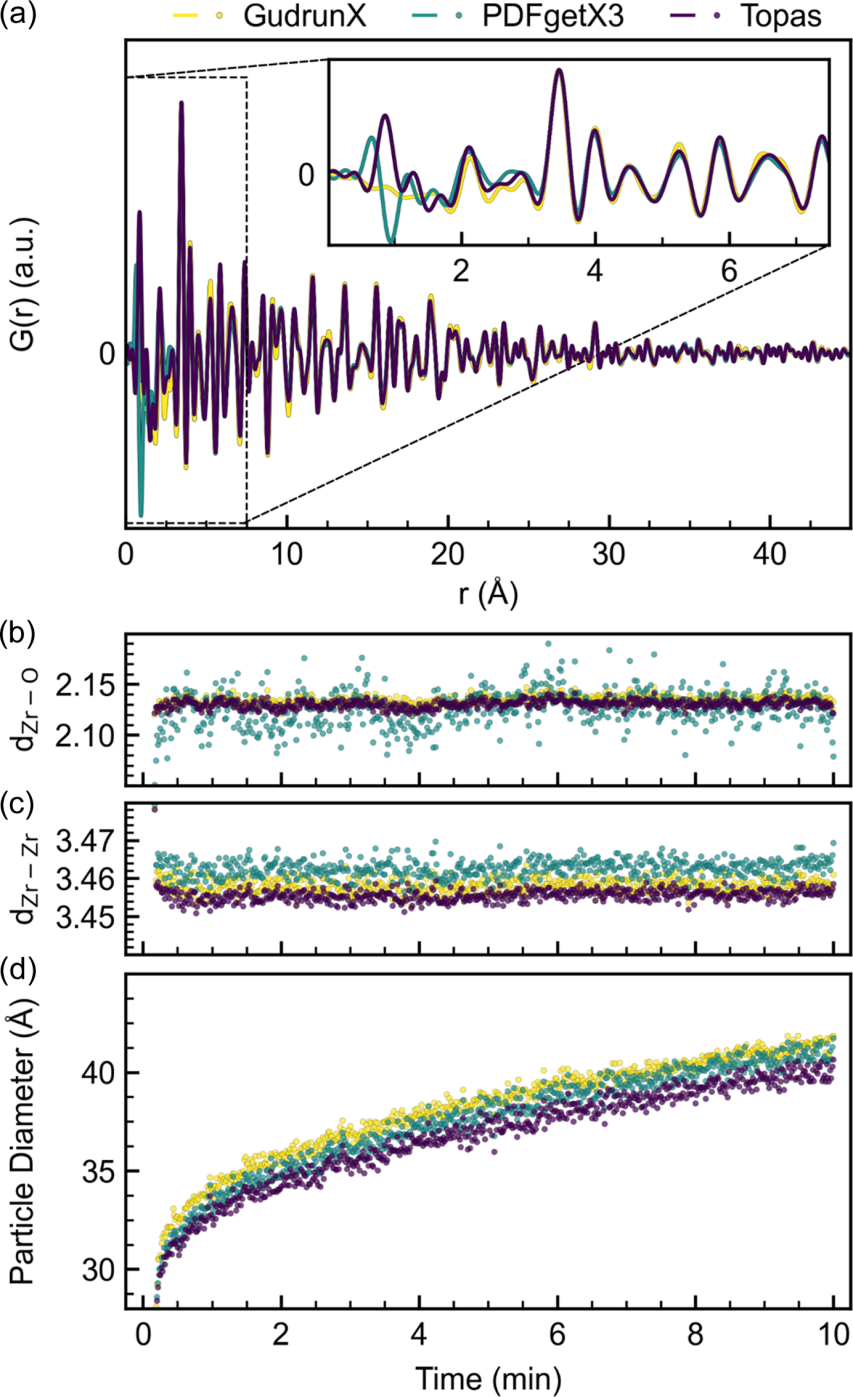
(*a*) Final PDF obtained from *in situ* experiment using *GudrunX*, *PDFgetX3* or *TOPAS*. (*b*) Zr—O bond distances, (*c*) Zr—Zr bond distances and (*d*) refined particle diameters as a function of time for the three different algorithms.

The PDFs obtained of the final frame, 10 min after the heating is applied, are shown in Fig. 8 ▸(*a*) and the corresponding *F*(*Q*) is shown in Fig. S26. Considering the raw PDFs, the major features above ∼4 Å are similar across the three algorithms. Below ∼4 Å, the three PDFs are different. In particular, the PDFs obtained from *PDFgetX3* and *TOPAS* show significant peaks at ∼1 Å, which are unphysical features introduced by unsatisfactory data reduction. The region of interest for most PDF experiments is the local correlations below ∼10 Å as medium-to-long range correlations are often more conveniently handled in reciprocal space. The major discrepancies across the three PDFs are observed below 1.5 Å, which in most inorganic solids would be below the shortest correlation expected and can thus be regarded as insignificant for the analysis. However, the introduction of unphysical features in the region of interest of the PDF could easily lead to overinterpretation, highlighting the significance of rigorous data reduction when analyzing the local correlations. Further analysis of the local correlations was performed by single peak fitting of the shortest Zr—O and Zr—Zr distances [Figs. 8 ▸(*b*) and 8 ▸(*c*)]. Both distances are found to be constant during the experiment, yielding a Zr—O distance of 2.12 Å and a Zr—Zr distance of 3.45–3.47 Å. Interestingly, the position of the Zr—O peak fluctuates by ∼0.1 Å when using the *PDFgetX3* algorithm, whereas the fluctuations observed using *GudrunX* or *TOPAS* are much smaller (<0.05 Å). The fluctuations could be a result of improper correction of the incoherent scattering using *R*_poly_ or could simply be a result of unintentional Fourier noise.

Sequential refinements of the time-resolved PDFs were performed for all algorithms. The results are summarized in Fig. S27, and the particle sizes obtained are plotted in Fig. 8 ▸(*d*). The growth phenomena observed are similar for the three algorithms, but the sizes obtained are offset by ∼3 Å at all times. This offset in particle sizes is probably due to small differences in background subtraction as shown in the analysis of the repetition data. The refined sizes [Fig. 8 ▸(*d*)] also show that the sizes obtained from *GudrunX* deviate less from the mean compared with the sizes obtained from *TOPAS* and *PDFgetX3*, which is most likely a result of reduced Fourier noise. The analysis shows that the choice of software used to obtain the PDFs clearly affects the resulting PDFs, but it does not seem to affect the obtainable information across different algorithms apart from a slight offset, mainly apparent for particle-size values. Due to the inclusion of Fourier noise, users should be aware of the risk of overinterpreting subtle features.

With both run-to-run variations and data processing affecting the results obtained from the PDF analysis, it is important to have common guidelines for drawing conclusions based on the PDFs. Run-to-run variations are almost impossible to eliminate but they should be kept in mind when interpreting the results. To enable comparisons between experiments, it is important to be consistent when generating the PDF; the same algorithm and the same parameters should be chosen for generating the PDF. However, background correction will likely still differ slightly between two different experiments, making it difficult to compare absolute values. Instead, conclusions should be drawn on the basis of relative trends. Systematically varying the input parameters for generating the PDF highlights the importance of complete transparency when reporting PDF results. Direct comparison with previously reported results is thus dependent on how the PDFs were calculated and deviations in absolute values should be expected.

## 4. Conclusions

The reproducibility of *in situ* solvothermal TS experiments was probed through the formation of ZrO_2_ nanoparticles from ZrCl_4_/H_2_O precursor solution. The extracted crystallite parameters and subsequent kinetic modeling display a high degree of reproducibility with less than 5 and 2% deviation in particle size and unit-cell volume, respectively. The low deviation is explained by very reliable sample positioning and good control over the experimental parameters across the entire beam time. We observe little change in the analysis when including data above *Q*_max_ = 18 Å^−1^, which is slightly below the recommended *Q*_max_ in the literature.

The parameters applied in the construction of the PDF from the experimental data were found to affect the extracted crystallite parameters with the variations being on the same scale as between individual experiments. The choice of software used to obtain the PDF was found to offset and change the variation of extractable parameters as well. The most prominent differences were found at low *r* values, explained by the different subtractions of inelastic scattering applied by the programs. Significant care should thus be taken when interpreting the low-*r* region of the PDF, as also pointed out by Egami & Billinge (2012 ▸). The relative trends in refined parameter values are largely unaffected by the run-to-run variation, the choice of data reduction parameters and the choice of software. This shows that observed trends are reliably obtained from *in situ* PDF analysis, and on the basis of such trends it should be possible to draw conclusions regarding the underlying chemistry of the nucleation and growth process of nanoparticles during solvothermal reactions. Absolute parameter values depend significantly on exactly how the PDFs were calculated, and therefore comparison with previously reported results should be done with care.

## Supplementary Material

Supporting figures. DOI: 10.1107/S1600576725001694/oc5043sup1.pdf
