# Supplementary material for: Reliability of pair distribution function analysis in in situ experiments
Source: J Appl Crystallogr. 2025 Mar 19;58(Pt 2):495–503. doi: 10.1107/S1600576725001694 (PMC11957418; doi:10.1107/S1600576725001694)
Supplement: Supplementary file 1 [file j-58-00495-sup1.pdf]

JOURNAL OF
APPLIED
CRYSTALLOGRAPHY

Volume 58 (2025)

Supporting information for article:

Reliability of pair distribution function analysis in *in situ* experiments

Rasmus Baden Stubkjær, Magnus Kløve, Andreas Bertelsen, Anders Bæk Borup, Martin Roelsgaard and Bo Brummerstedt Iversen

Contents

1) Temperature calibration 2
2) Variation in observed precursor 3
3) Overview of experiment in reciprocal space 4
4) Reduced structure function of all ten repetitions after 10 s 4
5) Sequential refinement results for ten repetitions 5
6) Result of Q_{damp} variation 7
7) Result of background variation: 9
8) Result of Q_{max} variation 10
9) Result of Q_{min} variation 12
10) Result of R_{poly} variation 14
11) Result of varying the number of bins during integration 16
12) Result of varying the composition 18
13) Result of varying the processing algorithm 19
14) Example of input file for PDF calculation using GudrunX 21
15) Example of input file for PDF calculation using Topas 23
16) Example of input file for PDF calculation using PDFgetX3 24

1) Temperature calibration

The temperature was calibrated at the beginning of the beamtime by inserting a thermocouple into the fused silica capillary. The tip of the thermocouple is placed at the point of the X-rays. The heater is calibrated to attain a smooth step heating curve at 250 °C with minimal overshot (Figure S1a). To find the correlation between the setpoint temperature of the heater and the temperature measured by the thermocouple several different temperatures are calibrated (Figure S1b).

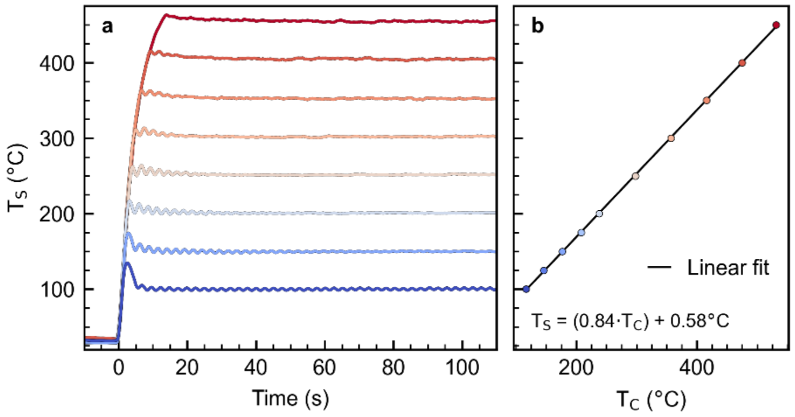

Figure S1. a) Temperature calibration for beamtime at P21.1 showing step heating from 100°C to 450°C in steps of 50°C. b) The linear correlation between the sample temperature, T_s, and the temperature control, T_c.

For eight out of ten of the *in situ* experiments performed at P21.1 the temperature control was logged for each frame (Figure S2). In rep. 1 and 2 the temperature was not logged; however, no major difference is expected for these two experiments.

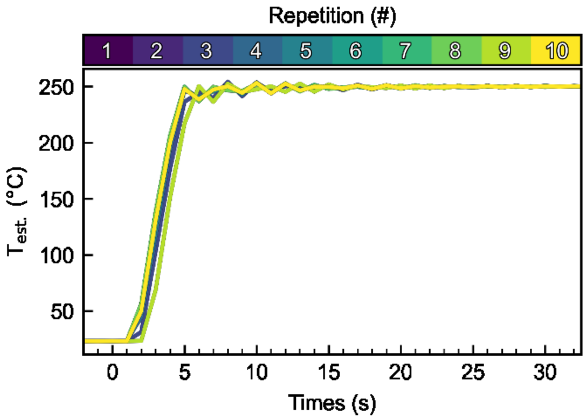

Figure S2. Estimated temperature of for repetition three to ten. The temperature is estimated from the linear correlation shown in Figure S1.

2) Variation in observed precursor

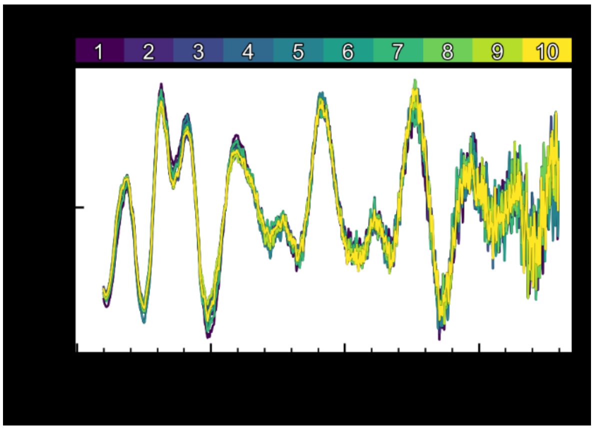

Figure S3. Variation in the reduced structure function, F(Q), of the precursors observed across all ten repetitions.

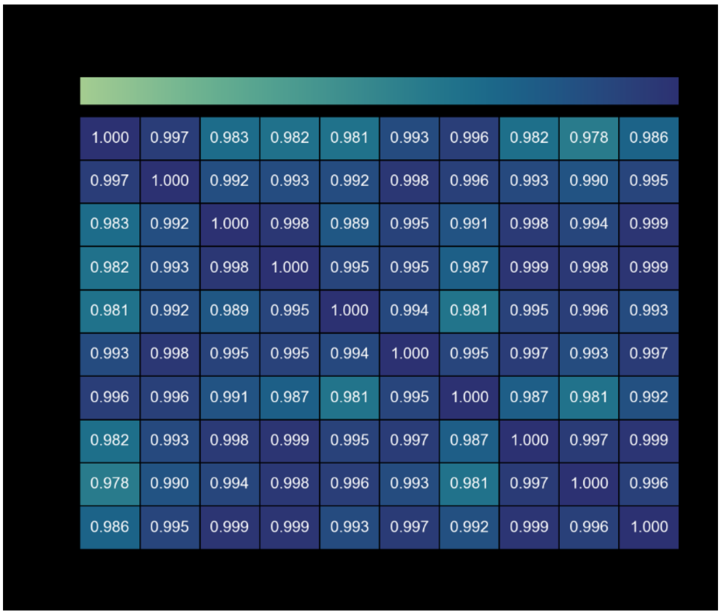

Figure S4. Pearson correlation matrix of the precursors observed across all ten repetitions

3) Overview of experiment in reciprocal space

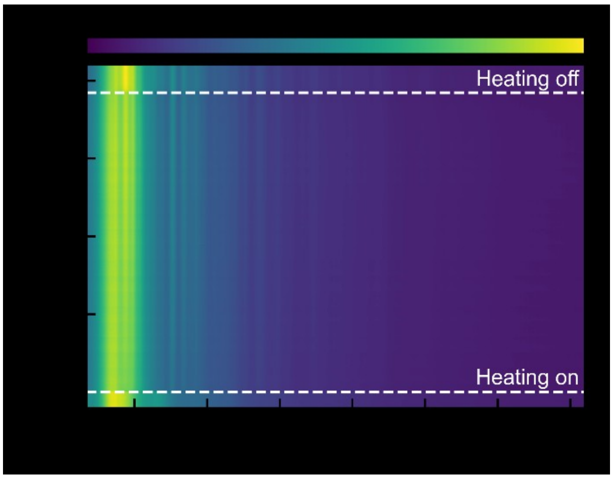

Figure S5. 2D contour plot of the time-resolved total scattering patterns for Rep. 1.

4) Reduced structure function of all ten repetitions after 10 s

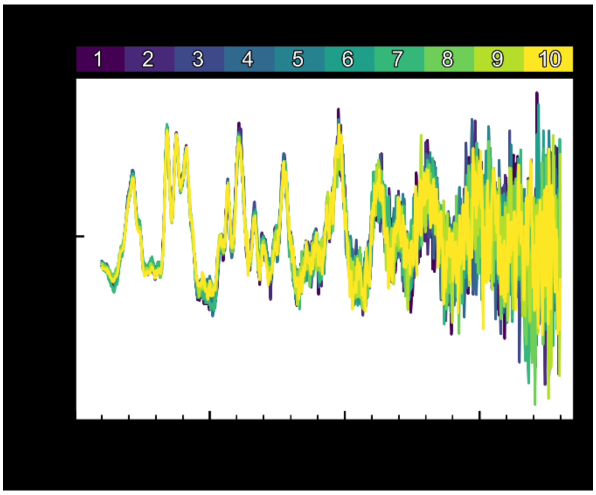

Figure S6. Variation in the reduced structure function, F(Q), approx. 10 s after the heat is applied across all ten repetitions.

5) Sequential refinement results for ten repetitions

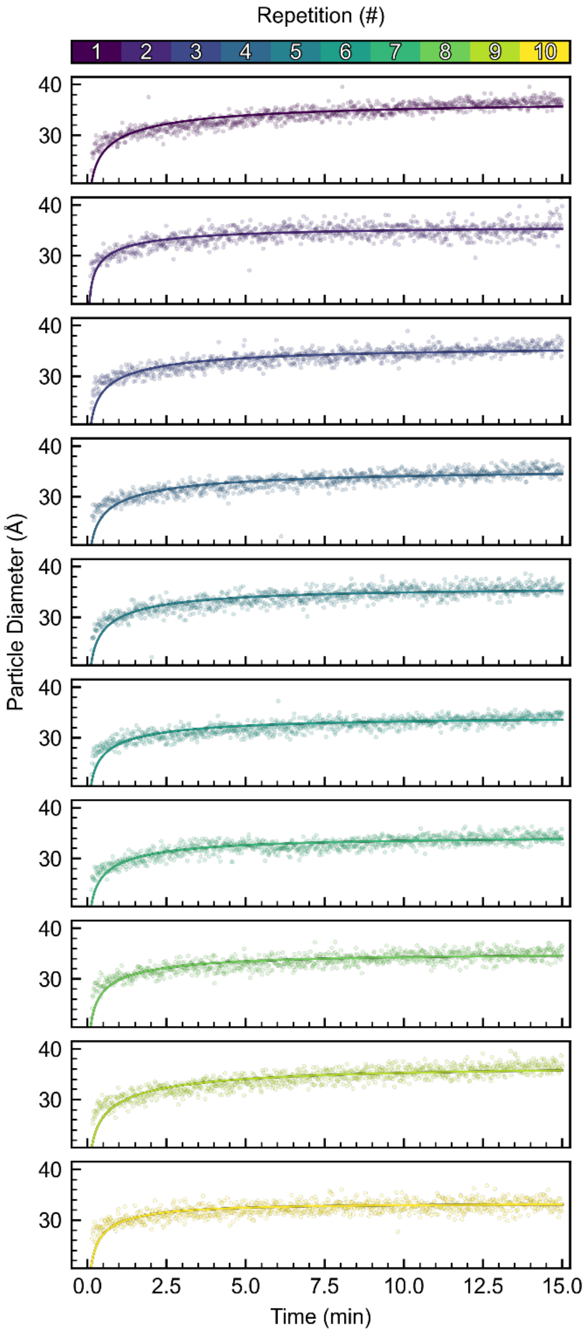

Figure S7. The particle diameter obtained from sequential refinements for each individual repetition. The fitted kinetic model (Eq. 1) is plotted on each growth curve.

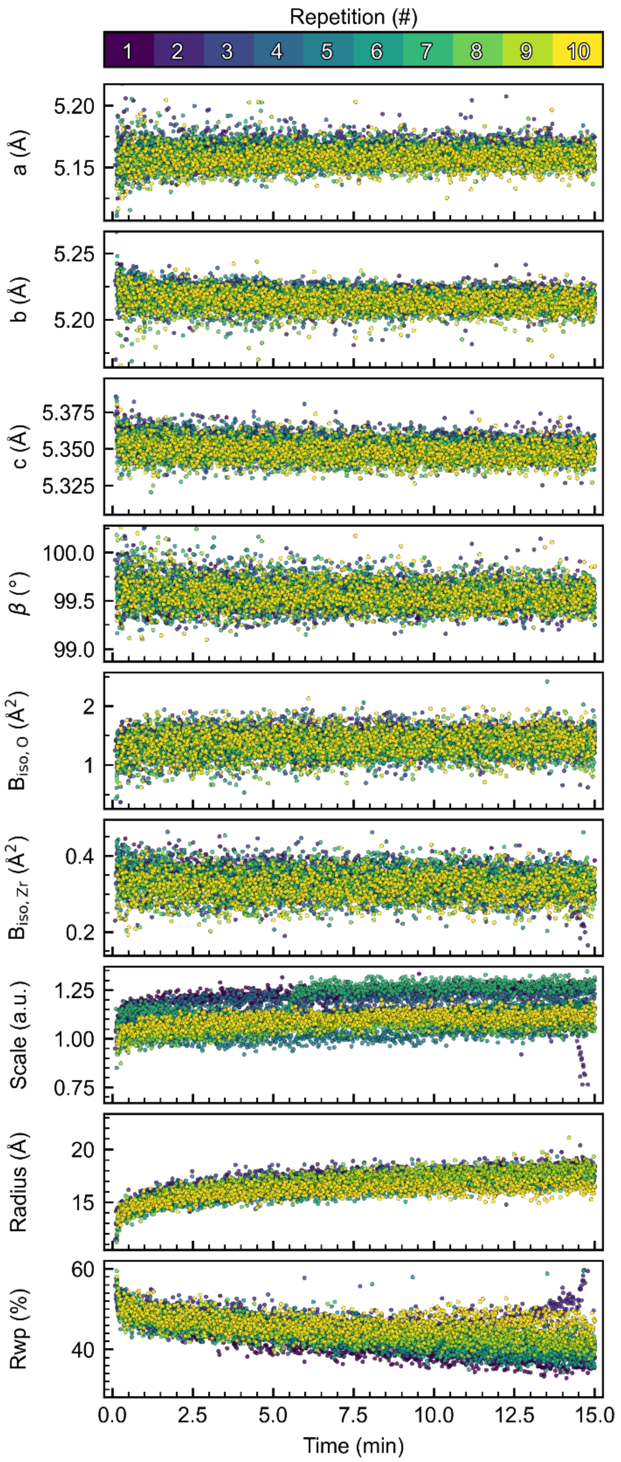

Figure S8. Result of the real-space sequential refinement. The refined parameters as a function of time for the ten repetitions.

6) Result of Q_{damp} variation

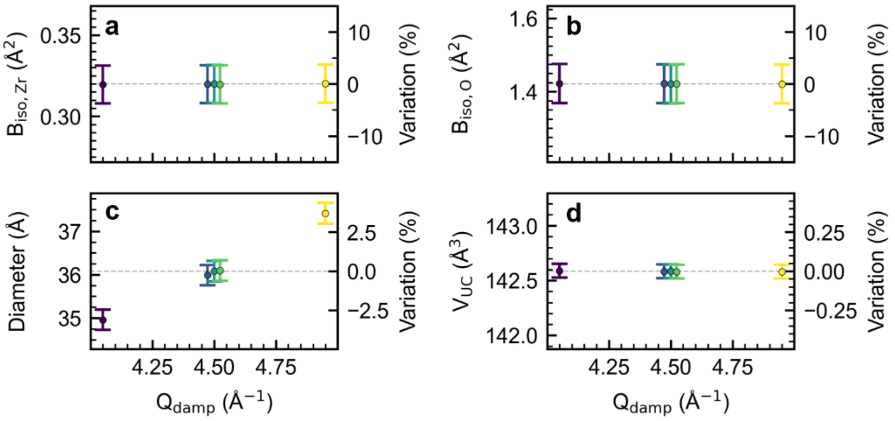

Figure S9. Variation of refined parameters during the final minute of the experiment. a) $B_{iso,Zr}$, b) $B_{iso,O}$, c) particle diameter, d) unit cell volume using varying Q_{damp} values. The baseline value used is Q_{damp} = 4.50 Å^{-1}.

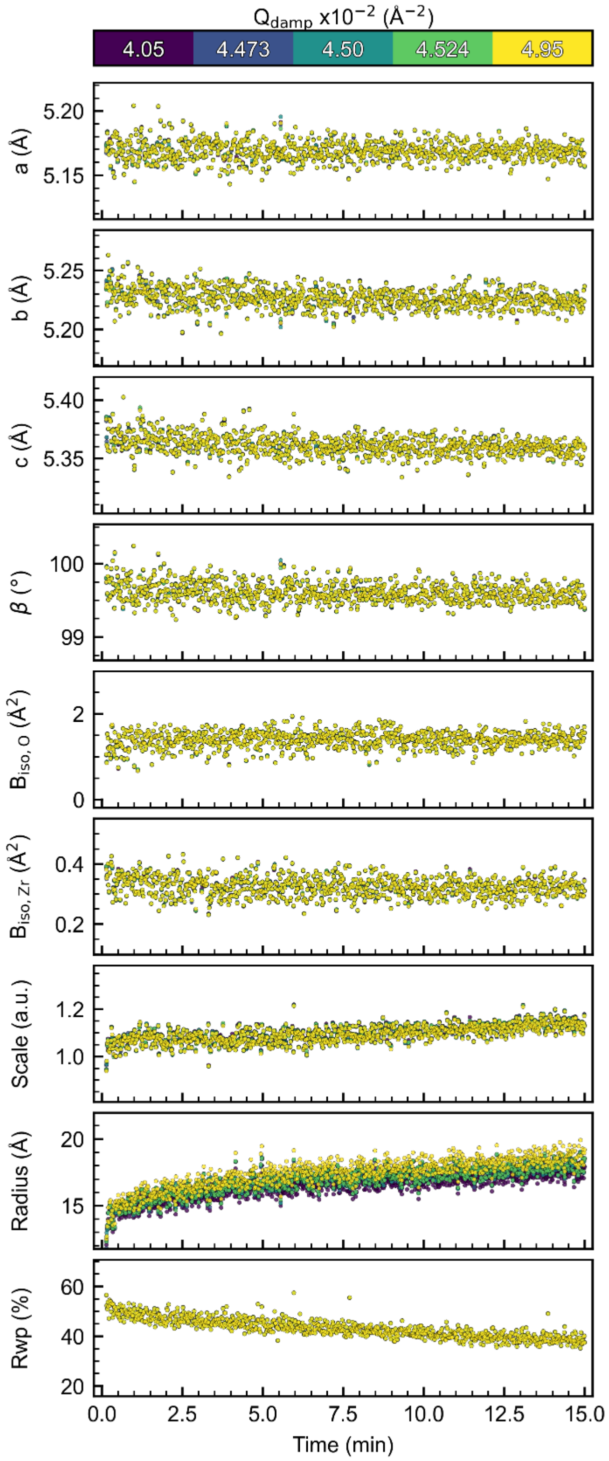

Figure S10. Result of the real-space sequential refinement using varying Q_{damp} values.

7) Result of background variation:

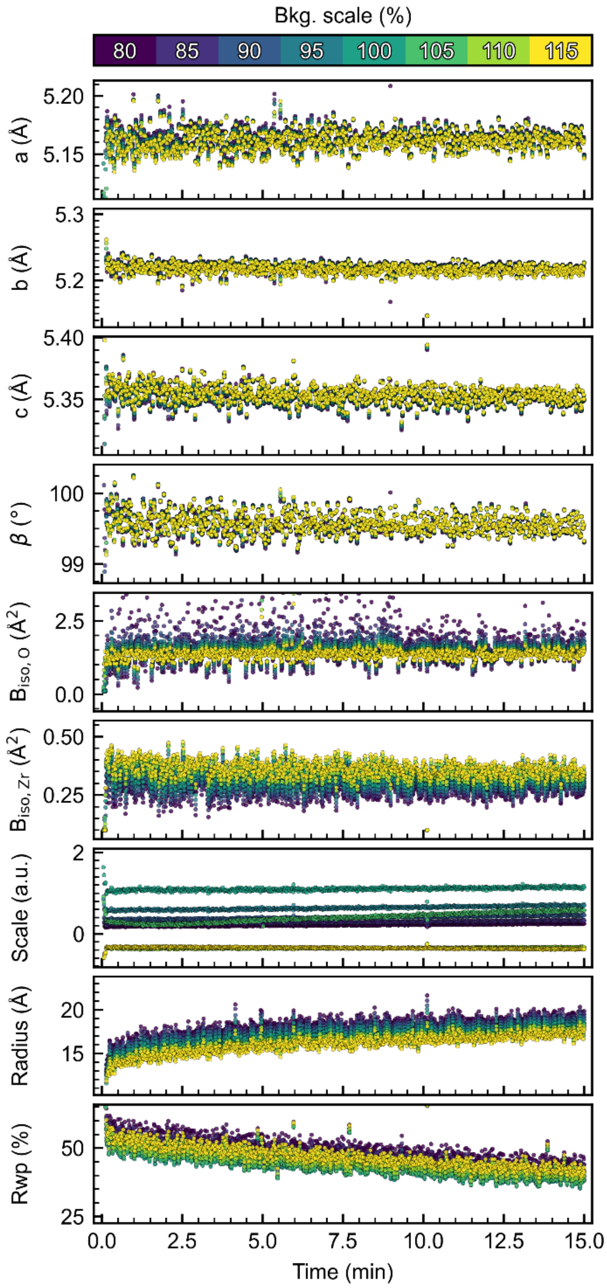

Figure S11. Result of the real-space sequential refinement using varying background scale.

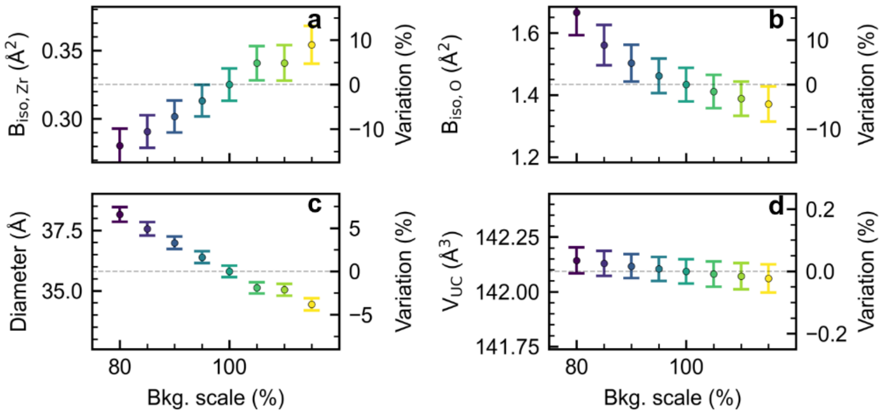

Figure S12. Variation of refined parameters during the final minute of the experiment a) $B_{iso,Zr}$, b) $B_{iso,O}$, c) particle diameter, d) unit cell volume) using varying background scale . The baseline value used is a background scale of 100%.

8) Result of Q_{max} variation

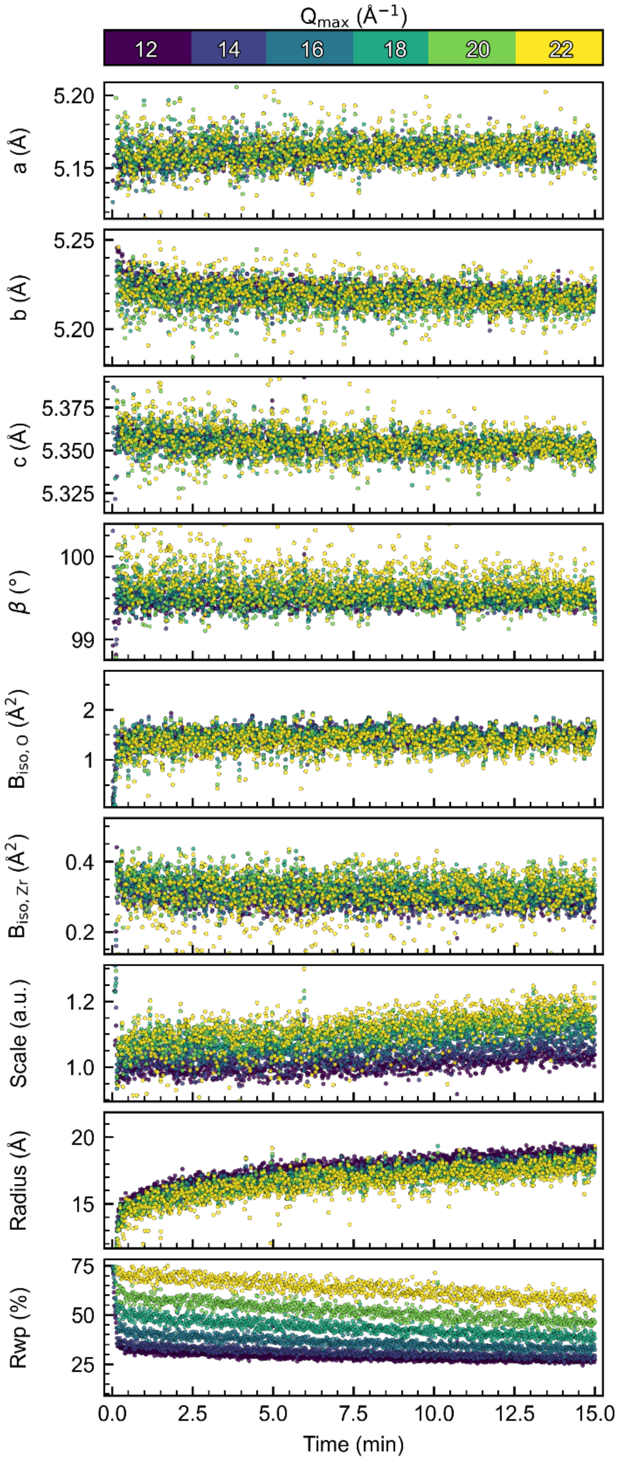

Figure S13. Result of the real-space sequential refinement using varying Q_{max} values.

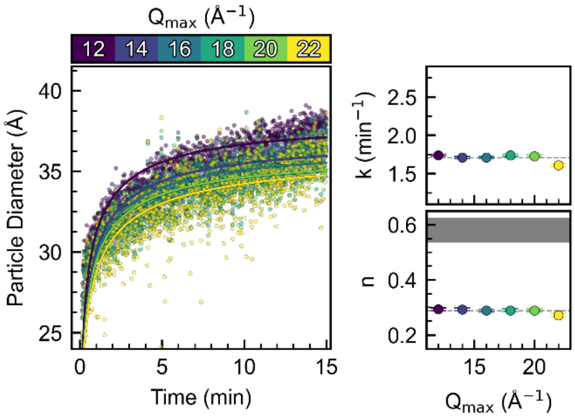

Figure S14. (Left) The obtained particle diameter obtained from sequential refinements using varying $Q_{\max}$ values. Fitted growth models (Eq. 1) are plotted on top of each growth-curve. (Right) Result of the fitted growth curve for each $Q_{\max}$ value.

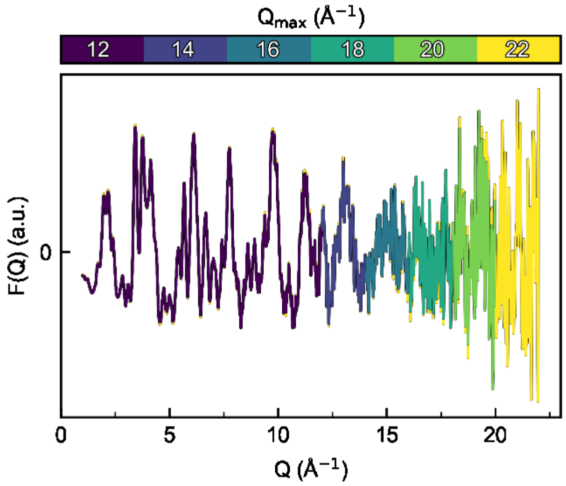

Figure S15. The reduced structure function, F(Q), obtained from *in situ* experiment after 15 min using varying $Q_{\max}$ values from 12 to 22 Å^{-1}.

9) Result of Q_{min} variation

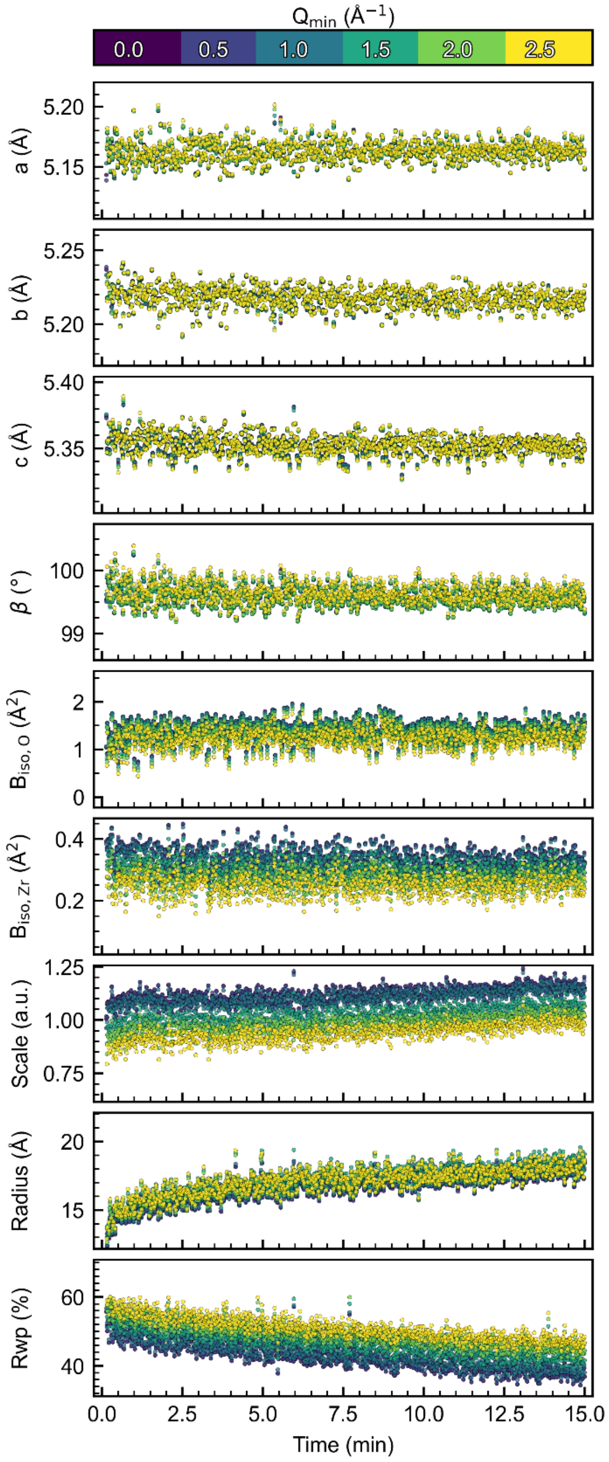

Figure S16. Result of the real-space sequential refinement using varying Q_{min} values.

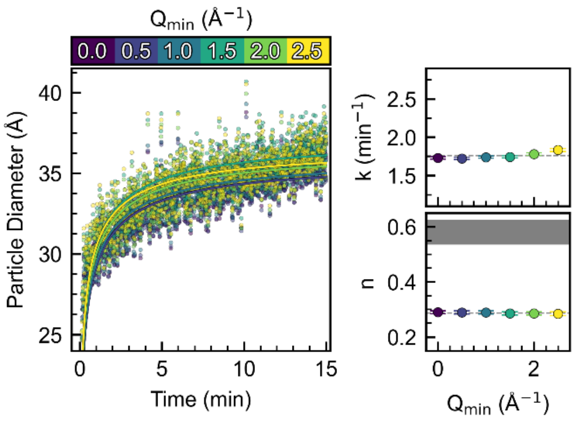

Figure S17. (Left) The obtained particle diameter obtained from sequential refinements using varying $Q_{\min}$ values. Fitted growth models (Eq. 1) are plotted on top of each growth-curve. (Right) Result of the fitted growth curve for each $Q_{\min}$ value.

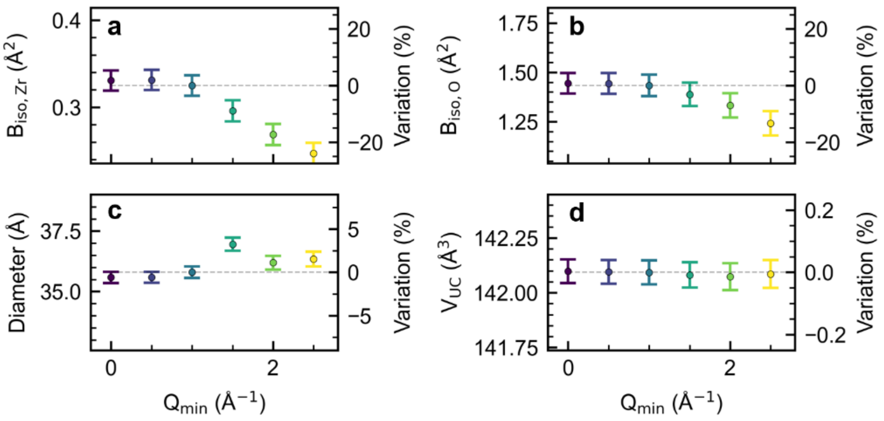

Figure S18. Variation of refined parameters during the final minute of the experiment. a) $B_{iso,Zr}$, b) $B_{iso,O}$, c) particle diameter, and d) unit cell volume using varying $Q_{\min}$ values. The baseline value used is $Q_{\min}$ = 1 Å^{-1}.

10) Result of R_{poly} variation

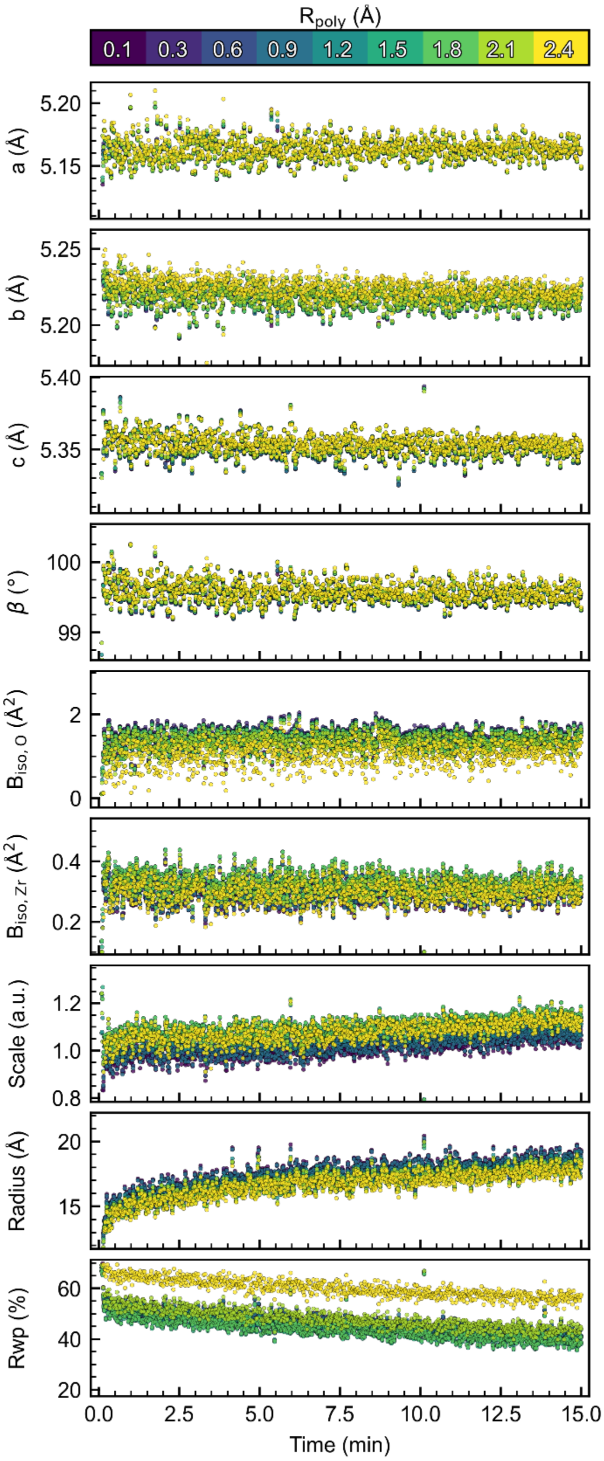

Figure S19. Result of the real-space sequential refinement using varying R_{poly} values.

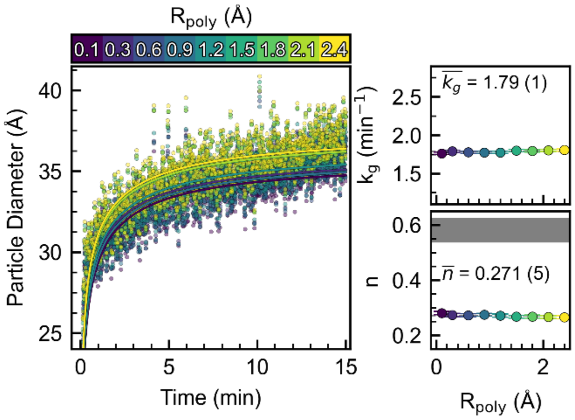

Figure S20. (Left) The obtained particle diameter obtained from sequential refinements using varying R_{poly} values. Fitted growth models (Eq. 1) are plotted on top of each growth-curve. (Right) Result of the fitted growth curve for each R_{poly} value.

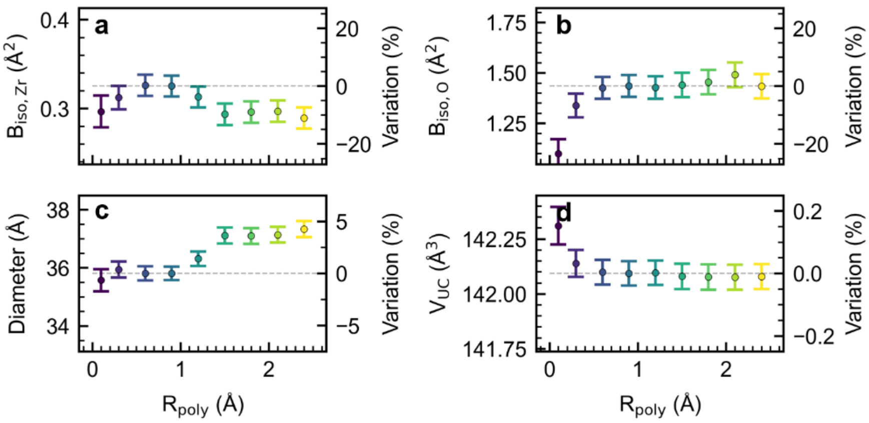

Figure S21. Variation of refined parameters during the final minute of the experiment a) $B_{\mathrm{iso,Zr}}$, b) $B_{\mathrm{iso,O}}$, c) particle diameter, and d) unit cell volume using varying R_{poly} values. The baseline value used is R_{poly} = 0.9 Å.

11) Result of varying the number of bins during integration

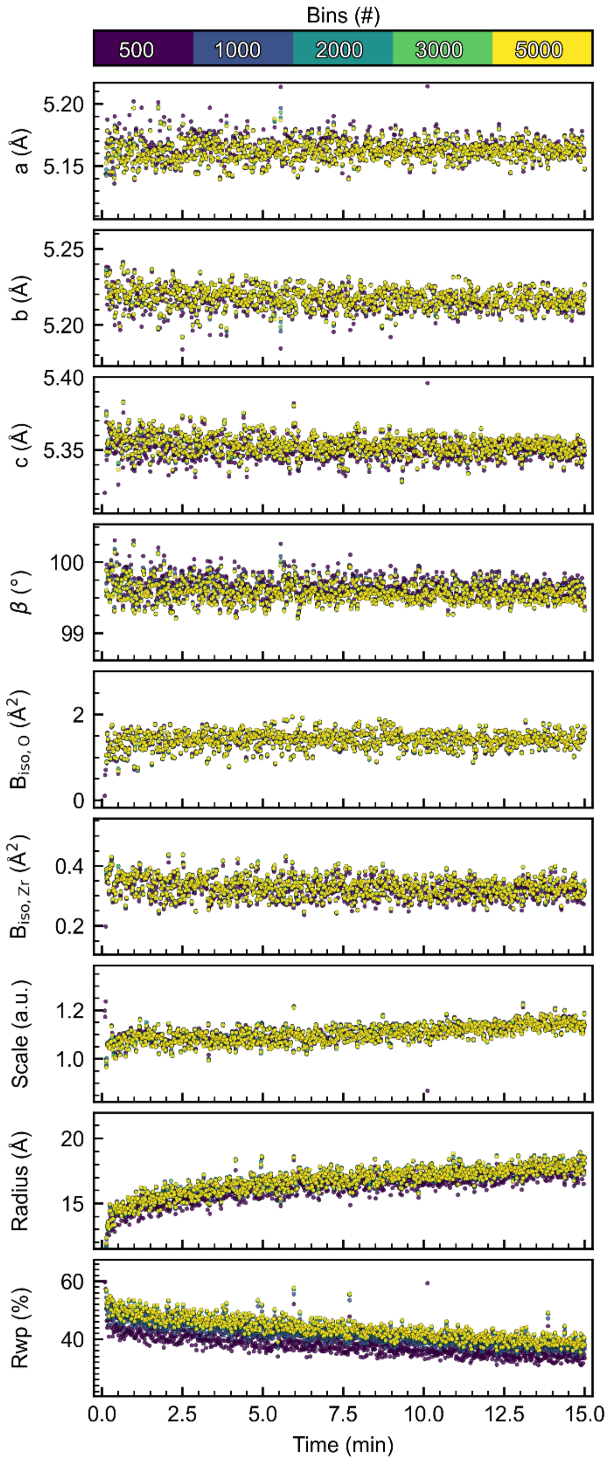

Figure S22. Result of the real-space sequential refinement using varying number of bins during azimuthal integration of 2D detector image.

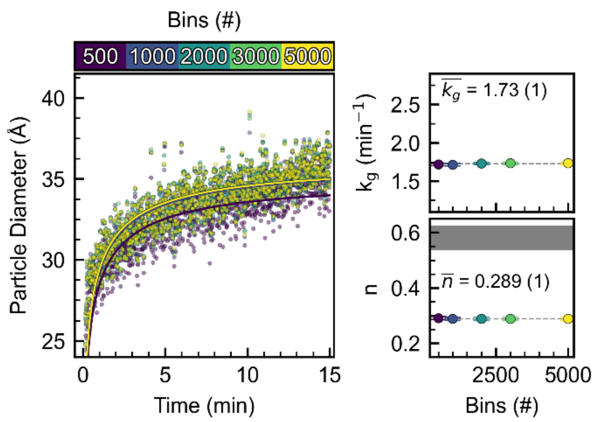

Figure S23. (Left) The obtained particle diameter obtained from sequential refinements using varying number of bins. Fitted growth models (Eq. 1) are plotted on top of each growth-curve. (Right) Result of the fitted growth curve for each number of bins used.

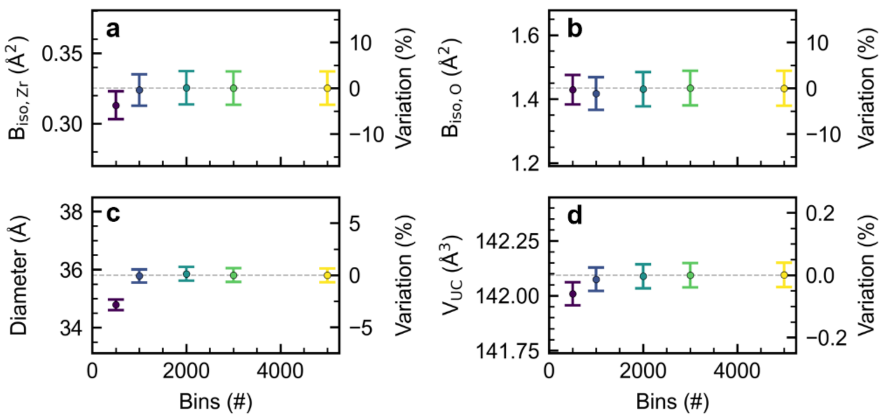

Figure S24. Variation of refined parameters during the final minute of the experiment a): $B_{iso,Zr}$, b) $B_{iso,O}$, c) particle radius, d) unit cell volume using varying number of bins. The baseline value used is 3000 bins.

12) Result of varying the composition

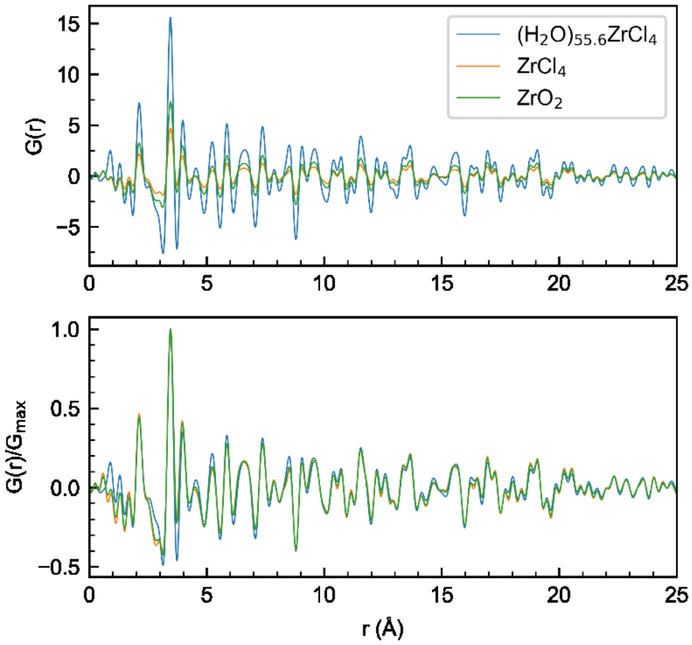

Figure S25. PDFs (top) and normalized PDFs (bottom) of the final frame of Rep. 1 obtained at approx. 15 min. The PDFs are calculated using three different compositions $(H_2O)_{55.6}ZrCl_4$ (blue), $ZrCl_4$ (orange), and ZrO_2 (green).

13) Result of varying the processing algorithm

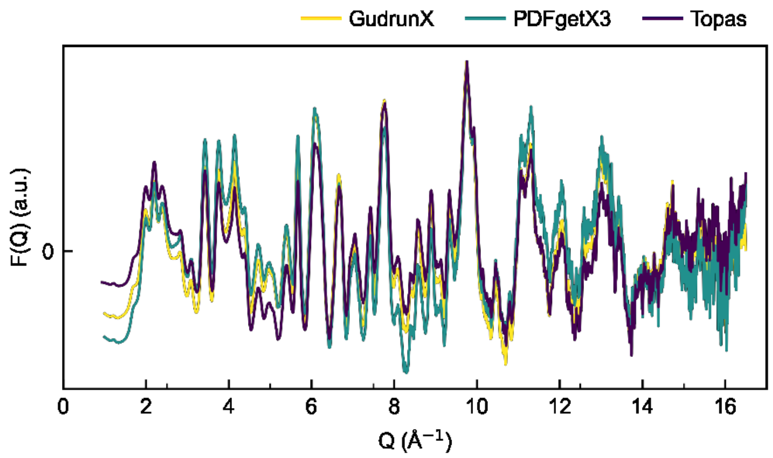

Figure S26. The reduced structure function, F(Q), obtained from *in situ* experiment after 15 min using either TOPAS v7, PDFgetX3 or GudrunX.

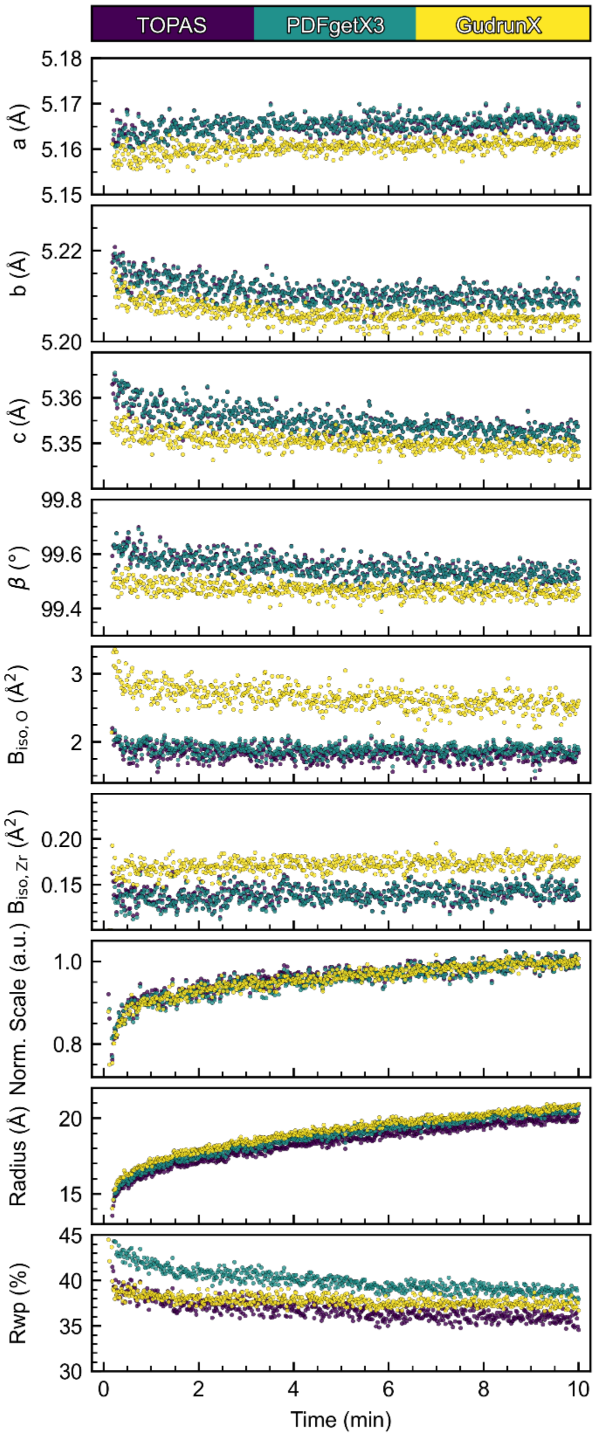

Figure S27. Result of the real-space sequential refinement using either TOPAS v7, PDFgetX3, or GudrunX to obtain the PDFs.

14) Example of input file for PDF calculation using GudrunX

```
'  '  '          '  '\'

INSTRUMENT          {

XRD          Instrument name
C:\Users\au565875\Documents\Reproducibility\PartC\GudrunX\Gudrun_input\          Gudrun input file directory:
C:\Users\au565875\Documents\Reproducibility\PartC\GudrunX\Data\ZrO2\          Data file directory
xy          Data file type
StartupFiles\Xray\CrossSec_XCOM.txt          X-ray cross sections file
StartupFiles\Xray\f0_WaasKirf.txt          X-ray form factor file
StartupFiles\Xray\CrossSec_Compton_Balyuzi.txt          X-ray Compton scattering file
1  16.5  0.01          Q-range [1/Å] for final DCS
80  0.01          r-max and r-step for final g(r)

}

BEAM          {

CYLINDRICAL          Sample geometry
2          Number of beam profile values
1.0  1.0          Beam profile values (Maximum of 50 allowed currently)
0.0014  0.0070  100          Step size for absorption and m.s. calculation and no. of slices
10          Step in scattering angle to calculate corrections at: [deg.]
-0.01  0.01  -0.01  0.01          Incident beam edges relative to centre of sample [cm]
-2.0  2.0  -2.1  2.1          Scattered beam edges relative to centre of sample [cm]
*          File containing bremsstrahlung intensity
10.5          Density of target material [gm/cm^3]
0.003          Effective target penetration depth [cm]
Rh          K-beta filter
12.4          K-beta filter density [gm/cm^3]
0          K-beta filter thickness [cm]
0.96          Bremsstrahlung power
36          Detector cutoff [keV]
3          Cutoff width [keV]
*          Lowest scattering angle
*          Highest scattering angle
*          Scattering angle step
0          Angle offset [deg.]
*          Anode material:
*          Tube voltage [kV]
0.35424          Wavelength [A]:
0          Theta-theta scanning?
0          Fixed slits?
0.0  DeprecatedSpace  -1.0          Beam width at normal incidence and sample depression [cm]:
0.0  0.0  -1.0          Position, width and power for low angle cutoff [deg]:
*          Tube current [mA]
*          kAlpha1 [A]
*          kAlpha2 [A]
*          kBeta [A]
0.0  0.00          kAlpha2 and kBeta relative intensities:
0.0          Bremsstrahlung scattering amplitude
10          No. of bremsstrahlung iterations

}

NORMALISATION          {

0.0          Azimuthal angle of detector above scattering plane:
0          Divide by <F>^2?
2          Power for Breit-Dirac factor (2 -3)
1          Krogh-Moe & Norman normalisation
0.0          Overlap factor

}
```

```

SAMPLE BACKGROUND          {

1            Number of  files
emptyBeamline_201122_scan-5846_sum_average.xy          SAMPLE BACKGROUND data files
1          Sample background factor
1.0          Data factor
0          Exclude scans

}

SAMPLE ZrO2_Frame_00030          {

1            Number of  files
PXRD_ZrCl4_H2O_250_a_scan-5305_integrated-00030.xy          SAMPLE ZrO2_Frame_00030 data files
1          Force calculation of sample corrections?
H  H  2.0  0.0  0.0          Composition
O  O  1.0  0.0  0.0          Composition
Zr  Zr  0.018  0.0  0.0          Composition
Cl  Cl  0.072  0.0  0.0          Composition
*  0  0  0  0          * 0 0 0 0 to specify end of composition input
SameAsBeam          Geometry
0.0  0.035          Inner and outer radii [cm]
0.1          Sample height [cm]
1.0          Density Units:  gm/cm^3?
TABLES          Total cross section source
1.0          Tweak factor
1.5          Top hat width (1/Å) for cleaning up Fourier Transform
1          Minimum radius for Fourier Transform [Å]
0.0          Width of broadening in r-space [A]
0  0          0   0          to finish specifying wavelength range of resonance
0.0  0.0  1.0          Exponential amplitude, decay [?] and stretch
8.807263e-03          Sample calibration factor
100          No. of iterations
0.0 0.0 0.0 0.0 0.0 0.0 0.0 0.0 0.0          Fluorescence levels
1.0          Factor to modify multiple scattering (0 - 1)
-1          Incident beam polarization factor (-1 -> +1)
0.7          Factor for Compton scattering
0.0          Bremsstrahlung scattering amplitude
10          No. of bremsstrahlung iterations
0          Broadening power
0.0  0.00          kAlpha2 and kBeta relative intensities:
1.0          Data factor
1          Analyse this sample?
0          Exclude scans

}

CONTAINER SiO2 1mm capillary          {

1            Number of  files
BG_FS_scan-5369_sum_average.xy          CONTAINER SiO2 1mm capillary data files
Si  Si  1.0  0.0  0.0          Composition
O  O  2.0  0.0  0.0          Composition
*  0  0  0  0          * 0 0 0 0 to specify end of composition input
SameAsBeam          Geometry
0.035  0.0425          Inner and outer radii [cm]
2          Sample height [cm]
-0.0663          Density atoms/Å^3?
TABLES          Total cross section source
1          Tweak factor
1.0          Data factor
0          Exclude scans

}

GO
```

15) Example of input file for PDF calculation using Topas

```
Include_PDF_Generate
num_runs 3
#prm find_Inorm_scaled = Run_Number == 0;
#prm ad_hoc_correction = Run_Number == 1;
#prm fourier_transform = Run_Number == 2;
#prm fit_pdf = Run_Number == 3;

macro Data_File           { Data/PXRD_ZrCl4_H2O_250_a_scan-5305_integrated-00630 }
macro Bkg_File           { BG_FS_scan-5369_sum_average }
macro EXT_ { xy }
macro wiggle(jiggle) { val_on_continue = Val + Rand(-jiggle*0.01*Val, jiggle*0.01*Val); }

prm  !N_ = 2+1+0.072+0.018; :  3.09`
macro fav_2  {(2/N_ *f0_H    + 1/N_ *f0_O    + 0.072/N_ *f0_Cl    + 0.018/N_ *f0_Zr)^2 } ' formula of unit cell
macro f2_av {2/N_ (f0_H)^2 + 1/N_ (f0_O)^2 + 0.072/N_ (f0_Cl)^2 + 0.018/N_ (f0_Zr)^2 }
macro f2_av_fav_2 {(f2_av)/(fav_2)}
macro Emission { lam  ymin_on_ymax 0.0001   la 1 lo 0.35424 lh 1 }

macro R_Max { 100 }
macro dR { 0.01 }
macro Num_Hats { 0 } ' Best smoothing function for speed and accuracy

macro tth_start {3}
macro tth_finish {59}
macro Qmin  {1}
macro Qmax {16.5}
prm !Bg_scale = 1;

#if (find_Inorm_scaled)
    iters 0
    user_y bg ##Bkg_File##.##EXT_
    user_y data ##Data_File##.##EXT_
    yobs_eqn I_norm.xy =  (data - Bg_scale*bg)/(fav_2);
        gui_reload
        min = tth_start;
    max =  tth_finish;
    del = 0.01;
    Emission
        xdd_sum I_norm_dot_f2avg = (Yobs*f2_av_fav_2); :  14907654.9`
        xdd_sum I_norm_dot_self = (Yobs^2); :  2.75409136e+09
        xdd_out Inorm_##Data_File##.##EXT_ load out_record out_fmt out_eqn { " %.9g  " = (4 Pi / Lam) Sin(X Pi / 360); " %.9g\n" =  (Yobs); }
#endif

#if (ad_hoc_correction)
    iters 100

    xdd Inorm_##Data_File##.##EXT_
        weighting =X/X2 ;'If(X > (X2 - (X2/4)), 2, 1);
        Emission
        rebin_with_dx_of 0.01
        No_Th_Dependence

        'Chebychev polynomial correction
        bkg bgl  763.975437`  712.678645`   35.8807354` 0 0 0

        xdd_array pbg = Get(bkg);
        gui_reload
        xdd_out Fq_##Data_File##.##EXT_ load out_record out_fmt out_eqn { " %.9g  " = X; " %.9g\n" =  (Yobs - Ycalc)* X; }
        xdd_out Poly_##Data_File##.##EXT_ load out_record out_fmt out_eqn { " %.9g  " = X; " %.9g\n" =  Ycalc; }
#endif

#if (fourier_transform)
        iters 0
        xdd Fq_##Data_File##.##EXT_
            rebin_with_dx_of 0.01
            start_X = Qmin;
            finish_X = Qmax;
            gui_reload
            pdf_generate {
            dr = dR;
            r_max = R_Max;
            gr_##EXT_##_file = String(Gr_##Data_File##);
            #if (Num_Hats)
                hat = Hat_Size; num_hats = Num_Hats;
            #endif
            }
#endif
```

16) Example of input file for PDF calculation using PDFgetX3

```
import os
import re
import numpy as np
from diffpy.pdfgetx import PDFGetter, PDFConfig, loadData

#Configurating PDFs
getPDF = PDFGetter()

### User input path! ###
background = r'\BG_FS\BG_FS_scan-5369_sum_average.xy'

###############################################################################################################################################

path = r'\Data/'

### Creating the dictionary with datafiles and folders as dict-keys ###

datafiles = []
for file in os.listdir(path):
    if file.endswith('.xy'):
        idx = re.findall('-(\d+).xy', file)[0]
        idx = int(idx)
        if idx >=30 and idx <= 630:
            datafiles.append(file)
        #else:
        #    print(idx)

datafiles.sort()

#################################################################################
###Configurating PDF
getPDF.config.wavelength = 0.35424 # Å
getPDF.config.backgroundfile = background
getPDF.config.bgscale = 1
getPDF.config.qmaxinst = 17.5
getPDF.config.qmax = 16.5
getPDF.config.qmin = 1
getPDF.config.composition = 'H2OZr0.018Cl0.072'
getPDF.config.rmax = 80
getPDF.config.rmin = 0
getPDF.config.rstep = 0.01
getPDF.config.rpoly = 0.9
getPDF.config.dataformat = 'twotheta'
getPDF.config.outputtypes = 'iq', 'sq','fq', 'gr'


##################################################################################
### Calculating PDF of just One dataset! ###
background = np.genfromtxt(background, skip_header =0)

getPDF.config.bgscale = 1



#print("Plotting: {}".format(datafiles_dict[key][::4][-50]))
single_frame = np.genfromtxt(path+datafiles[-1])
getPDF(single_frame[:,0],single_frame[:,1])

Iq = getPDF.iq
Sq = getPDF.sq
Fq = getPDF.fq
Gr = getPDF.gr
```
